# Identification of novel lipid biomarkers in *xmrk*- and *Myc*-induced models of hepatocellular carcinoma in zebrafish

**DOI:** 10.1186/s40170-022-00283-y

**Published:** 2022-04-04

**Authors:** Jerry D. Monroe, Daniel Fraher, Xiaoqian Huang, Natalie A. Mellett, Peter J. Meikle, Andrew J. Sinclair, Seth T. Lirette, Nita J. Maihle, Zhiyuan Gong, Yann Gibert

**Affiliations:** 1 Department of Cell and Molecular Biology, Cancer Center and Research Institute, University of Mississippi Medical Center, Jackson, MS 39216 USA; 2 Metabolic Genetic Diseases Laboratory, Metabolic Research Unit, Deakin University School of Medicine, 75 Pigdons Road, Geelong, VIC 3216 Australia; 3 Department of Biological Sciences, National University of Singapore, Singapore, Singapore; 4 Baker Heart and Diabetes Institute, 75 Commercial Road, Melbourne, VIC 3004 Australia; 5 Department of Nutrition, Dietetics and Food, Monash University, Notting Hill, VIC 3168 Australia; 6 Department of Data Science, University of Mississippi Medical Center, Jackson, MS 39216 USA

**Keywords:** Zebrafish, Cancer, Liver, Lipids, Lipidomics, Hepatocarcinoma, *Myc*, *xmrk*, Transgenic oncogene models

## Abstract

**Background:**

Hepatocellular carcinoma (HCC) is the predominant form of liver cancer and is accompanied by complex dysregulation of lipids. Increasing evidence suggests that particular lipid species are associated with HCC progression. Here, we aimed to identify lipid biomarkers of HCC associated with the induction of two oncogenes, *xmrk*, a zebrafish homolog of the human epidermal growth factor receptor (EGFR), and *Myc*, a regulator of EGFR expression during HCC.

**Methods:**

We induced HCC in transgenic *xmrk*, *Myc*, and *xmrk*/*Myc* zebrafish models. Liver specimens were histologically analyzed to characterize the HCC stage, Oil-Red-O stained to detect lipids, and liquid chromatography/mass spectrometry analyzed to assign and quantify lipid species. Quantitative real-time polymerase chain reaction was used to measure lipid metabolic gene expression in liver samples. Lipid species data was analyzed using univariate and multivariate logistic modeling to correlate lipid class levels with HCC progression.

**Results:**

We found that induction of *xmrk*, *Myc* and *xmrk*/*Myc* caused different stages of HCC. Lipid deposition and class levels generally increased during tumor progression, but triglyceride levels decreased. *Myc* appears to control early HCC stage lipid species levels in double transgenics, whereas *xmrk* may take over this role in later stages. Lipid metabolic gene expression can be regulated by either *xmrk*, *Myc*, or both oncogenes. Our computational models showed that variations in total levels of several lipid classes are associated with HCC progression.

**Conclusions:**

These data indicate that *xmrk* and *Myc* can temporally regulate lipid species that may serve as effective biomarkers of HCC progression.

**Supplementary Information:**

The online version contains supplementary material available at 10.1186/s40170-022-00283-y.

## Introduction

The adult liver can regulate lipid synthesis and degradation allowing it to play a major role in lipid metabolism [1]. Dysregulation of lipid production occurs in human hepatocellular carcinoma (HCC) [2, 3], a form of liver cancer that is the third leading cause of cancer-related death in the world according to the World Health Organization (https://www.who.int/news-room/fact-sheets/detail/cancer). Because of the societal impact of HCC, there is considerable interest in the development of new models that can act as analytical platforms for the study of this disease. The zebrafish model is readily adaptable for studying human cancer as the zebrafish genome exhibits a high degree of sequence conservation with human oncogenes, exhibits similar tumor development and physiology, and is readily adaptable to genetic and chemical screening [4, 5]. Further, cancer cells expressed in zebrafish have very similar genetic and genomic characteristics relative to their human counterparts [6]. Recently, transgenic adult zebrafish models have been successfully used to study oncogenes, immune physiology, gender-based effects, hormonal signaling, metastasis, drug efficacy, and tumor progression and regression in HCC [7–13].

We recently generated a doxycycline (DOX)-inducible liver tumor zebrafish model that transgenically expresses the myelocytomatosis (*Myc*) and the *Xiphophorus* melanoma receptor tyrosine kinase (*xmrk* activated epidermal growth factor receptor (EGFR) homolog) oncogenes [10, 11]. These models allowed us to control the temporality and location of oncogene induction via DOX exposure through regulation of the liver fatty acid–binding protein (*fabp10a*) promoter which is only expressed in hepatocytes [14]. Here, we used the *xmrk* and *Myc* transgenic fish models to identify potential lipid biomarkers of HCC progression. Histological analysis demonstrated that *Myc* fish developed hyperplasia, *xmrk* fish induced HCC stage I, and some *xmrk/Myc* fish acquired the characteristics of HCC stage II. Single and double transgenic fish had less lipid accumulation in the early stages of tumor progression but began to exhibit markedly increased lipid deposition in the later stages. This increased lipid accumulation is similar to conditions observed during nonalcoholic fatty liver disease (NAFLD), a spectrum of disorders that comprise nonalcoholic steatohepatitis, cirrhosis, and HCC [15], although specific lipid levels can be either decreased or increased in NAFLD [16].

Comparative lipidomic analysis of the different transgenic models revealed that the levels of most lipid species in cancer cells increased except for triglycerides. Examination of the lipid species regulation profiles suggests that *Myc* may drive certain changes in lipid levels at earlier time points and that *xmrk* may take over this role at some later stage before the onset of mortality. Analyses of the relative expression of lipogenic transcription factors, lipogenic enzymes, and lipid β-oxidation genes indicate that *xmrk* and *Myc* may regulate distinct metabolic genes and may even counteract each other’s effects. Univariate and multifactorial modeling showed that several lipid classes increased during HCC progression, but that increased levels of other classes were correlated with restoration of the normal phenotype. Thus, *xmrk* and *Myc* signaling may act through specific lipids and lipid metabolic genes which may have utility as either diagnostic or prognostic biomarkers.

## Methods

### Zebrafish maintenance and transgene induction

Zebrafish embryos were raised as previously described [17]. Adult zebrafish were euthanized by adding 1× tricaine (Sigma, St. Louis, USA) to the maintenance solution. Generation of *TO(xmrk)* (x+m−); *TO(Myc)* (x−m+) and *TO(xmrk/Myc)* (x+m+) transgenic models under the control of the *lfabp* promoter has been described previously [8, 11]. All zebrafish studies were approved by the Deakin University Animal Welfare Committee (AWC G17-2015) and the Institutional Animal Care and Use Committee of the National University of Singapore (Protocol 079/07). Transgenes were induced by exposing adult zebrafish (90 days old) to 40 μg/ml doxycycline (DOX) [10, 11].

### Histology sample preparation and analysis

Liver samples taken from 1.5, 3, 4.5, 6, and 7.5 days post-DOX treatment (dpt) fish were slowly dehydrated using a series of increasing ethanol solution concentrations (70%, 90%, 95%, and 100%). Specimens were then embedded in paraffin using a Leica EG1120. Sectioning at 5 μm was performed using a Reichert-Jung 2030 microtome. Hematoxylin (H) (Vector Laboratories, Burlingame, CA, #H3404) and Eosin (E; Sigma, #HT110232) were used for H and E staining. Oil-Red-O (Sigma) staining of zebrafish liver sections was performed as previously described [18]. Identification and classification of tumor types were based on previously established criteria [6].

### Lipidomic analysis

To normalize samples, three to seven zebrafish livers were dissected and homogenized using a Pro200 homogenizer (Pro Scientific, Oxford, CT). Protein concentration was then quantified using a Pierce™ BCA protein assay kit (Life Technologies, Carlsbad, CA). An aliquot of each sample normalized to 30 μg was used for lipid extraction. Samples and standards used in this analysis were as previously described [19]. Briefly, samples from 1.5, 3, 4.5, 6, and 7.5 dpt were lyophilized to remove all liquid. Samples were then reconstituted prior to extraction in 10 μl of water and 10 μl of the internal standard mix was added to each sample. Lipids were extracted by adding 200 μl chloroform/methanol (2:1) followed by sonication for 30 min before the supernatant was transferred to a 96-well plate and dried under vacuum in a SpeedVac Concentrator (Thermo Scientific, Waltham, MA). Samples were then reconstituted with 50 μl water-saturated butanol and 50 μl methanol with 10 mM ammonium formate, and analyzed by LC ESI-MS/MS, a technique that allows consistent measurement of various lipid species and classes in zebrafish over different experimental time points [20], using an Agilent 1200 LC system (Santa Clara, CA) and an AB Sciex 4000 Qtrap mass spectrometer (Darmstadt, Germany). Data were analyzed using MultiQuant 2.1 software (AB Sciex).

### Quantitative real-time PCR analysis

Total RNA was extracted from 3, 4.5, and 6 dpt liver samples using the TRIzol® (Invitrogen #15596-018) and chloroform method per the manufacturer’s protocol. RNA was reverse-transcribed into cDNA using Transcriptor First Strand cDNA Synthesis Kit (Roche Applied Science, Penzberg, Germany). qRT-PCR was carried out using SYBR Green I Master Mix (Roche Applied Science) and a LightCycler® 480 machine (Roche Life Science). Cycle numbers (*C*_t_) of triplicate samples were averaged and then normalized to β-actin expression to obtain a Δ*C*_t_ value. Final gene expression levels were calculated as 2^−ΔΔCt^ (see Supplemental Table 1 for a complete list of primers used in this study).

### Lipid disease stage progression modeling analysis

As a final step, we constructed a model that would allow us to graphically depict the correlation between lipid levels and disease stage [21, 22]. 24 univariate ordinal logistic regression models relating each lipid to disease stage, i.e., normal, hyperplasia/adenoma, HCC1, and HCC2 were constructed using data from all time points (1.5, 3, 4.5, 6, 7.5 dpt). To facilitate ease of interpretation, each lipid was scaled by dividing the value by roughly ¼ of its standard deviation. Then, we put all 24 logistic regression models into a multivariate fractional polynomial (MFP) ordinal logistic model with backward selection variable procedures enabled. This approach allowed us to construct a concise model where only random error and no systematic bias is present in lipid measurement and where additive effects that might propagate are accounted for by removing lipids that contribute to multicollinearity and only those total lipid classes that display independence in each HCC stage are considered [20–22]. All variables remained linear after the MFP procedure, with the exception of GM3, which was given a square root transformation.

### Statistical analysis

Lipidomic samples were statistically evaluated (GraphPAD PRISM, La Jolla, CA) using an ANOVA with Tukey’s post hoc test. qRT-PCR samples were statistically evaluated using an ANOVA. All statistical analyses for the logistic modeling analysis were performed with Stata v16.1 (College Station, TX). A probability level of *p* < 0.05 was deemed significant throughout.

## Results

### Rapid oncogenic transformation of hepatocytes into HCC cells in xmrk/Myc transgenic zebrafish

**Fig. 1:**
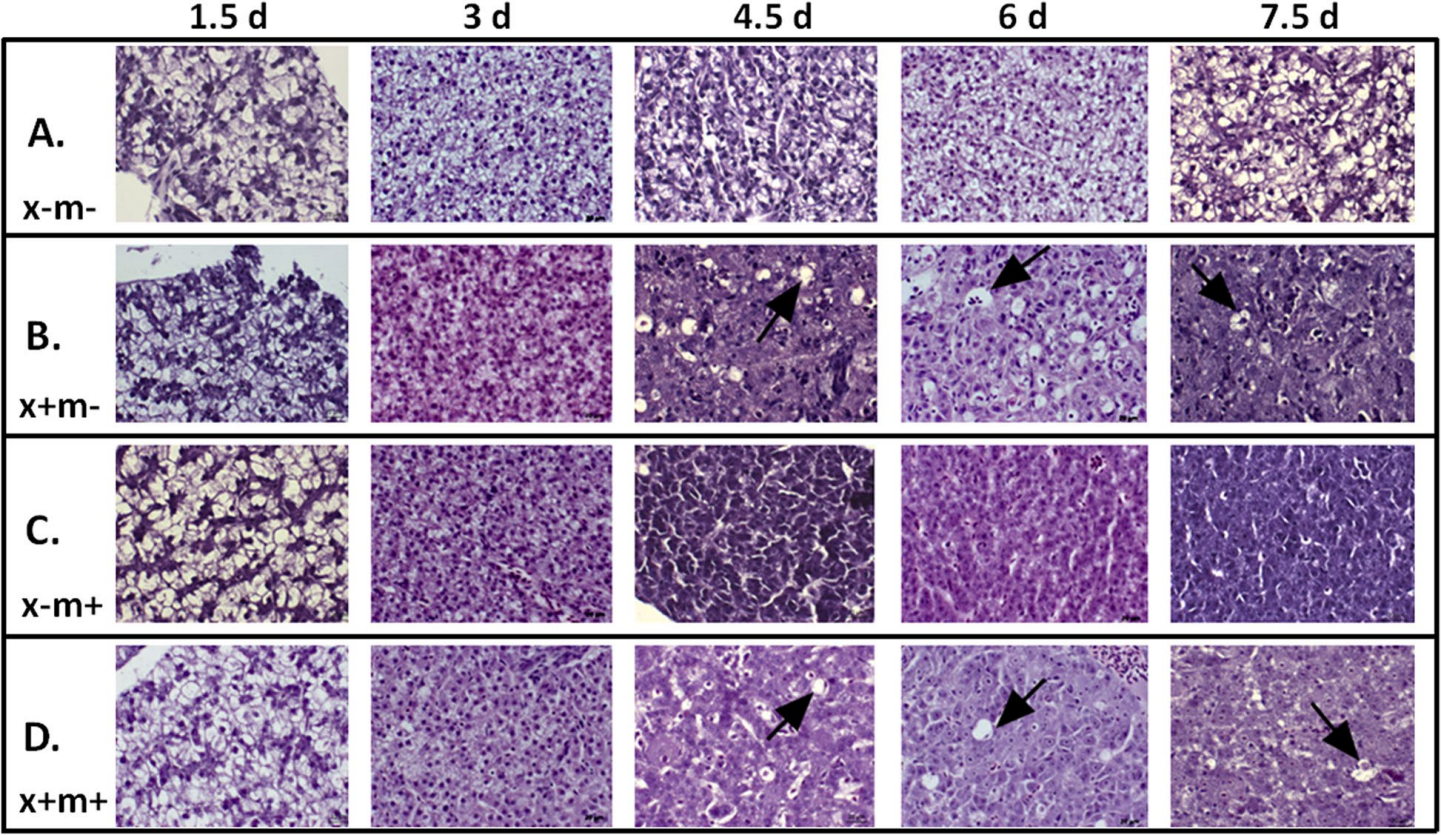
Induction of transgenes causes rapid oncogenic transformation of normal zebrafish liver cells into HCC cells. Representative histological cross sections of 3-month post-fertilization (mpf) DOX-treated zebrafish liver tissue from 1.5 to 7.5 dpt. Images were taken at 40× magnification. **A** x−m−, non-transgenic control; **B** x+m−, *xmrk* oncogene expression; **C** x−m+, *Myc* oncogene expression; **D** x+m+, *xmrk* and *Myc* oncogene expression. Key: black arrows, apoptotic cells; black boxes, syncytial cells with multiple nuclei indicating abnormal mitosis. Error bars = 20 μm

Three DOX-inducible zebrafish transgenic oncogene lines, *xmrk* (x+m−), *Myc* (x−m+), and *xmrk*/*Myc* (x+m+) were evaluated for their ability to transform hepatocytes into HCC cells. After 1.5 days post-treatment (dpt), all the transgenic groups (*N* = 10 per group) showed similar histology characteristics compared to the non-transgenic DOX-exposed control group (x−m−) (Fig. 1), but at 3 dpt, single (x+m− and x−m+) and double transgenic zebrafish (x+m+) exhibited hyperplasia with increased cell number (Fig. 1B–D). Cellular transformation to HCC Grade I was first detected at 4.5 dpt in x+m− and x+m+ samples with evident increased apoptosis (Fig. 1B, D, arrows), and multiple nuclei in one cell (Fig. 1D, black box). By 7.5 dpt, two x+m+ individuals showed grade II HCC phenotype, and x+m+ fish started to die after 7.5 dpt; therefore, 7.5 dpt was chosen as the end point for analysis. Unlike the other samples, x−m+ livers remained hyperplastic throughout all time points (Fig. 1C).

### Lipid class and species levels in zebrafish livers during tumor progression

**Fig. 2:**
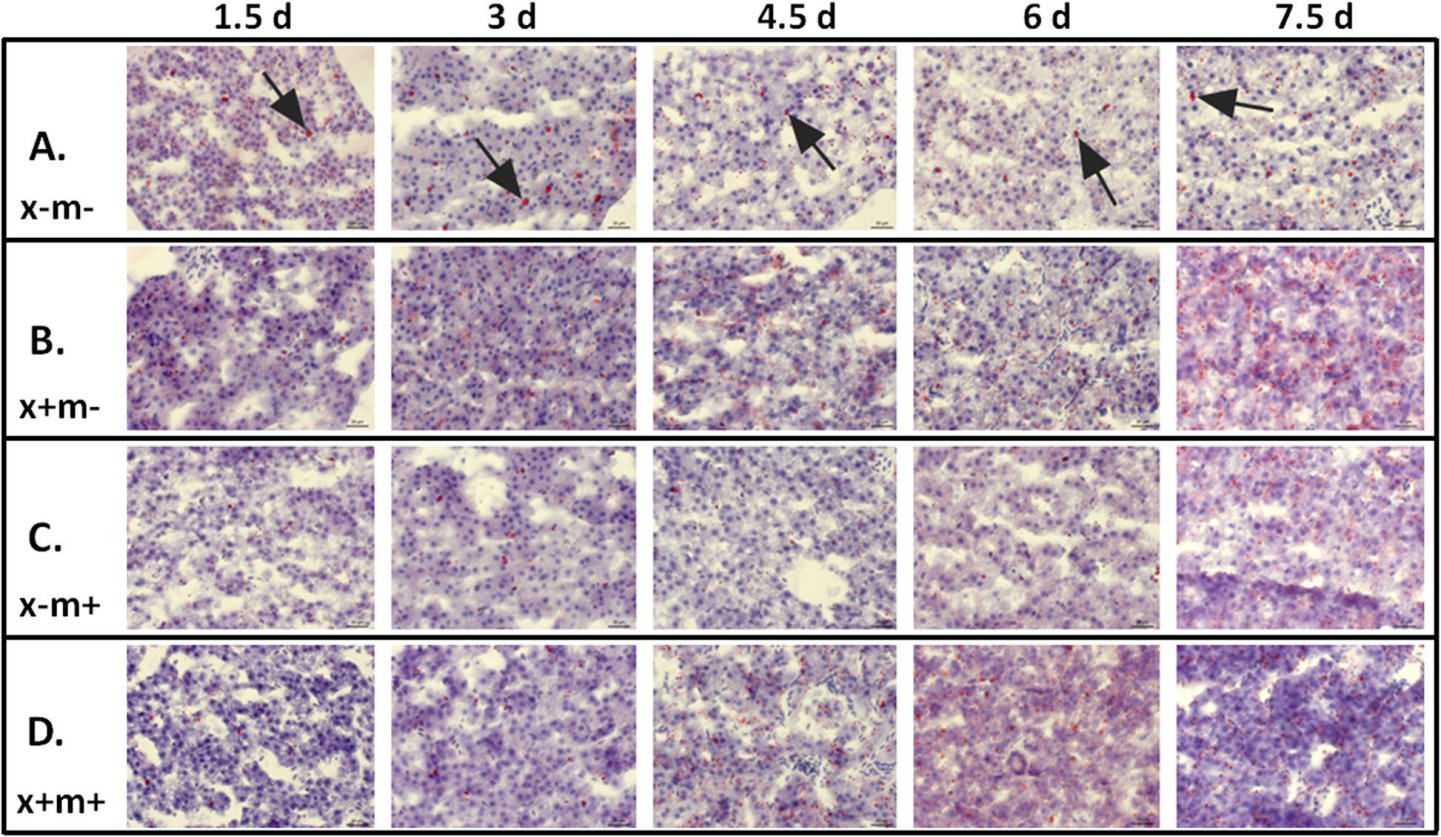
Lipid levels increase in zebrafish liver HCC tissue. Representative images of Oil-Red-O stained zebrafish tissue taken from three mpf DOX-treated zebrafish (1.5 to 7.5 dpt). Lipid droplets are stained in red and nuclei are counterstained in blue. **A** x−m−, non-transgenic control; **B** x+m−, *xmrk* oncogene expression; **C** x−m+, *Myc* oncogene expression; **D** x+m+, *xmrk* and *Myc* oncogene expression. Key: black arrows indicate large lipid droplets. Error bars = 20 μm

**Fig. 3:**
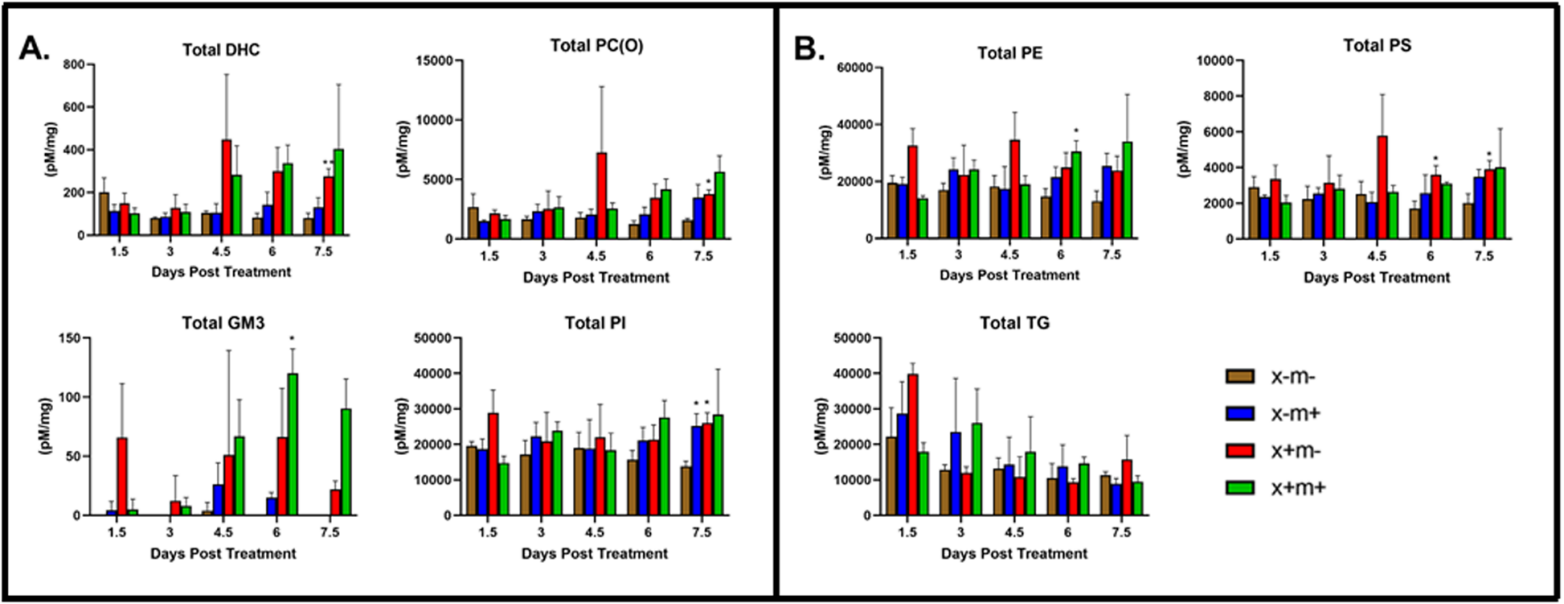
Total lipid class abundance during HCC transgene expression. Many lipid classes exhibited altered levels in three mpf DOX-treated zebrafish tissue samples from 1.5 to 7.5 dpt. **A** DHC, PC(O), GM3, and PS levels. **B** PE, PI, and TG levels. Color key: x−m−, brown column; x−m+, blue column; x+m−, red column; x+m+, green column. *p < 0.05; **p < 0.01

**Fig. 4:**
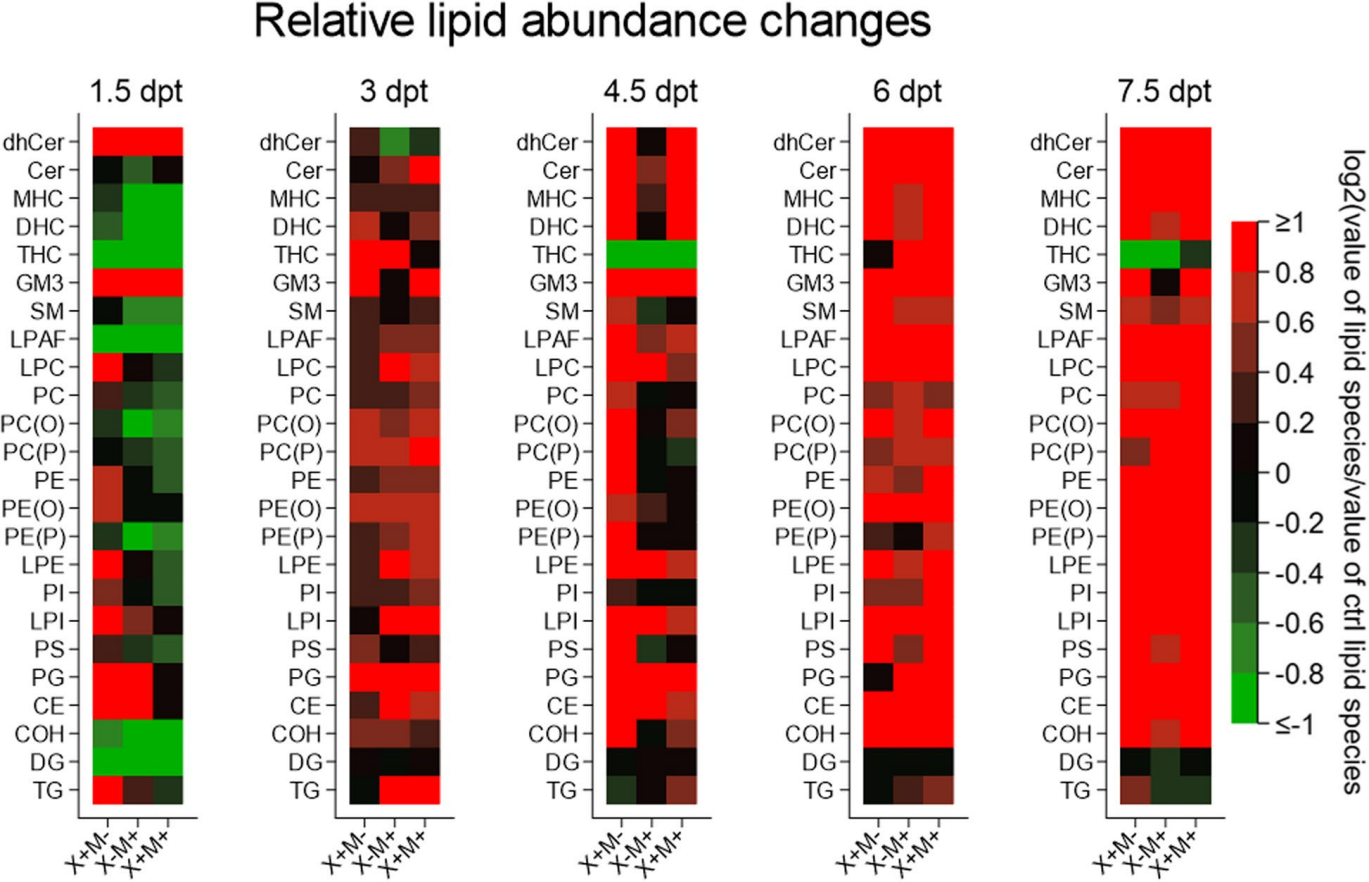
Relative lipid class abundance changes in zebrafish liver tumor tissue. Abundance of 24 lipid classes in three mpf DOX-treated zebrafish from 1.5 to 7.5 dpt (see text for lipid class abbreviation definition). Key: x+m−, *xmrk* oncogene expression; x−m+, *Myc* oncogene expression; x+m+, *xmrk* and *Myc* oncogene expression; red (upregulated), black (unchanged), green (downregulated). Data expressed as log2 transformation of value of lipid species/value of control lipid species

Oil-red-O (ORO) staining was performed on liver samples to detect lipids. We found that x−m− samples had red staining lipid droplets present across all time points (1.5 to 7.5 dpt), while in transgenic samples, droplets were also detected and became particularly noticeable at 7.5 dpt (Fig. 2). Interestingly, lipid droplets are detected in greater number in control x−m− shortly after DOX induction (1.5 dpt to 3 dpt); however, after 6 and 7.5 dpt, lipid droplets are present in excess in all three transgene models compared to controls (Fig. 2). We analyzed liver sample lipid levels from 1.5 to 7.5 dpt using LC ESI-MS/MS. We identified many lipid classes whose levels exhibited temporal changes during these intervals, e.g., triglycerides (TG), dihexosylceramide (DHC), alkylphosphatidylcholine (PC(O)), monosialodihexosyl-ganglioside (GM3), dihydroceramides (dhCer), ceramides (Cer), monohexosylceramide (MHC), trihexosylceramide (THC), sphingomyelin (SM), lyso-platelet-activating factor (LPAF), lysophosphatidylcholine (LPC), phosphatidylcholine (PC), alkenylphosphatidyl-choline (PC(P)), phosphatidylethanolamine (PE), alkylphosphatidylethanolamine (PE(O)), alkenylphosphatidylethanolamine (PE(P)), lysophosphatidylethanolamine (LPE), phosphatidylinositol (PI), lysophosphatidylinositol (LPI), phosphatidylserine (PS), phosphatidylglycerol (PG), cholesteryl ester (CE), free cholesterol (COH), and diacylglycerol (DG) (Fig. 3, supplementary Fig. 1). DHC and PC(O) levels in x+m− fish were higher at 7.5 dpt (Fig. 3A). GM3 lipid levels in x+m+ samples were increased at 6 dpt (Fig. 3A). PI lipid levels in x+m− and x−m+ samples were significantly higher at 7.5 dpt (Fig. 3A). At 6 dpt, x+m+ livers expressed higher levels of PE (Fig. 3B), and at 6 and 7.5 dpt, x+m− samples had higher levels of PS (Fig. 3B). TG levels exhibited a downward trend across all time points (Fig. 3B). Heat-map analysis revealed that relative lipid class abundance in the transgenic tissues compared to control generally increased between 1.5 to 7.5 dpt (Fig. 4).

The LC ESI-MS/MS analysis also showed that inducing *xmrk* and *Myc* caused altered levels of numerous lipid species (Table 1, supplementary Fig. 2). In x−m+ samples, we found the following levels at various time points: 1.5 dpt, several decreased PEs, PE 20.4-0:0 increased; 3 dpt, PC(O)s, PEs, and PI 20:4-0-0 increased; 4.5 dpt PC(O) 35:4 was increased; 6 dpt, PC(O) 20:0-0:0, PEs, and two PIs increased; 7.5 dpt, several TGs were decreased, while several PC(O)s, PEs, PIs, and PSs were all increased (see Table 1 for details). In x+m− samples, we found that *xmrk* induced regulation of different lipid species when compared to *Myc* transgenic samples. At 1.5 dpt, x+m− fish did not have decreased lipid species, but x+m− fish had increased levels of DHC 20:0, GM3 16:0, and multiple PC(O)s, PEs, PIs and TGs (Table 1). At 3 dpt, x+m− fish livers showed decreased levels of lipid species, e.g., the TGs, while only one PC(O), 35:4, was increased in x+m− samples (Table 1). *Xmrk* only transgenic samples at 4.5 dpt also showed increased PC(O) 35:4 and many other lipid species, e.g., several other PC(O)s, DHC 22:0, several PEs, PIs, and two PS species (Table 1). At 6 dpt, x+m− fish also showed increased PC(O) 20:0-0:0, but x+m− fish showed increased levels of DHCs, GM3 24:1, additional PC(O)s, PEs, PIs, and several PSs (Table 1). At 7.5 dpt, x+m− fish samples did not show decreased TGs, but did show increased levels of PC(O)s, PEs, PIs, and PSs, and *xmrk* induction alone increased DHCs (Table 1).

**Table 1:** Lipid species exhibiting altered levels during HCC

Transgene (dpt)	Lipid species
**X-/M+ (1.5)**	Dec: Cer22:0; Cer24:0; MHC24:0; SM33:1; SM34:0; SM35:1; SM38:1; SM39:1; SM41:1; SM41:2; SM42:1; PC31:1; PC31:0; PC32:0; PC33:3; PC33:2; PC33:0; PC34:3; PC35:2; PC36:6; PC36:5; PC37:6; PC37:4; PC38:7; PC39:7; PC39:6; PC39:5; PC40:8; PC-P 34:3; **PE36:5; PE36:4; PE36:2**; PE-O 18:2-22:5; PE-P 18:0-22:5; COH
	Inc: dhCer16:0; PC-P 30:0; **PE 20:4-0:0**; CE16:2; CE18:0; CE18:2; CE18:3; CE20:1; CE22:5; **TG14:1-16:0-18:1; TG16:0-16:0-18:2; TG14:0-18:0-18:1**
**X+/M- (1.5)**	Dec: SM41:2; PE-P 18:0-22:5; COH
	Inc: dhCer16:0; MHC20:0; **DHC20:0**; **GM3 16:0**; SM36:1; LPC15:0; LPC17:1; LPC18:0; LPC18:1; LPC20:2; LPC20:3; LPC24:0; PC36:6; PC36:1; PC38:5; PC38:2; PC40:4; **PC-O 34:4; PC-O 34:3; PC-O 36:4**;**PE32:1; PE34:3; PE34:1; PE35:2; PE35:1; PE36:3; PE36:1; PE40:6; PE40:5; PE 20:4-0:0**;**PI34:1; PI36:1; PI36:4; PI38:5; PI38:6; PI40:5; PI:18:0-0:0; PI:18:1-0:0; PI:20:4-0:0**; PG18:1-18:1; DG14:0-14:0; **TG14:1-16:0-18:1; TG18:1-14:0-16:0; TG15:0-18:1-16:0; TG17:0-16:0-16:1; TG17:0-18:1-14:0; TG14:0-18:2-18:2; TG14:1-18:0-18:2; TG14:1-18:1-18:1; TG16:1-16:1-18:1; TG16:0-16:0-18:2; TG16:1-16:1-18:0; TG16:0-16:1-18:1; TG14:0-18:0-18:1; TG16:0-16:0-18:1; TG15:0-18:1-18:1; TG17:0-18:1-16:1; TG17:0-18:2-16:0; TG17:0-18:1-16:0; TG16:0-18:2-18:2; TG16:1-18:1-18:2; TG16:1-18:1-18:1; TG16:0-18:1-18:2; TG16:0-18:1-18:1; TG16:0-18:0-18:1; TG17:0-18:1-18:1; TG18:1-18:2-18:2; TG18:0-18:2-18:2; TG18:2-18:2-20:4; TG18:1-18:1-20:4;TG18:1-18:1-22:6**
**X+/M+ (1.5)**	Dec: Cer22:0; MHC24:0; SM33:1; SM35:1; SM38:1; SM41:1; SM41:2; SM42:1; PC33:3; PC33:2; PC34:4; PC34:3; PC35:3; PC36:6; PC36:5; PC36:3; PC36:2; PC37:6; PC37:5; PC37:4; PC38:7; PC38:5; PC38:2; PC39:7; PC39:6; PC39:5; PC40:8; **PC-O 36:4; PC-O 40:7; PC-O 40:6**; PC-P 34:2; **PE36:5; PE36:5; PE36:4; PE36:3; PE36:2; PE38:5; PE38:4; PE38:3; PE 16:0-0:0**; **PI38:4**; **PS38:4**; COH; DG18:1-18:3
	Inc: dhCer16:0; **PI34:1**; CE16:2; CE18:2; **TG14:0-18:0-18:1**
**X-/M+ (3)**	Dec: SM36:1
	Inc: MHC24:0; SM33:1; LPC22:0; **PC-O 32:0; PC-O 18:0-0:0**; PC-P 34:2; **PE38:6; PE38:3; PE40:6; PE40:5; PE 18:1-0:0**; PE-O 40:6; PE-P 18:0-22:5; **PI:20:4-0:0**; CE18:0; CE20:2; CE22:4; CE22:5
**X+/M- (3)**	Dec: DG14:0-14:0; **TG16:1-16:1-16:1; TG16:1-16:1-18:0; TG16:0-16:1-18:1; TG18:2-18:2-20:4**
	Inc: **PC-O 35:4**
**X+/M+ (3)**	Dec: None decreased
	Inc: Cer22:0; Cer24:0; MHC24:0; PC40:4; **PC-O 32:0**; PC-P 38:5; **PE38:6; PE40:6; PE40:5**; PE-O 40:6; PE-P 16:0-22:6; **PI38:4**; CE22:4; CE24:5; **TG14:0-16:0-18:2; TG14:1-16:0-18:1; TG17:0-18:1-14:0; TG14:1-18:0-18:2; TG14:0-18:0-18:1; TG16:0-16:0-18:1; TG16:0-18:2-18:2**
**X-/M+ (4.5)**	Dec: PC34:5; PC34:4; PC35:5; PC36:6; PC38:7; PC-P 34:2; CE24:0
	Inc: PC38:3; **PC-O 35:4**; CE16:0; CE16:1; CE17:0; CE17:1; CE18:0; CE18:1; CE18:3; CE20:1; CE20:2; CE20:3; CE20:4; CE22:4; CE22:5; CE24:5; CE24:6
**X+/M- (4.5)**	Dec: PC-P 34:2
	Inc: MHC18:0; **DHC22:0**; SM37:2; SM38:2; LPC16:1; LPC17:1; LPC18:1; LPC18:2; LPC18:3; LPC22:0; PC:18:1-18:3; PC31:1; PC32:2; PC32:1; PC33:2; PC33:1; PC33:0; PC34:3; PC34:2; PC34:1; PC35:1; PC35:0; PC36:3; PC36:2; PC36:1; PC37:4; PC38:3; **PC-O 32:2; PC-O 34:3; PC-O 35:4; PC-O 36:3; PC-O 36:2**; PC-P 40:6; **PE34:3; PE34:2; PE34:1; PE35:1; PE36:3; PE36:2; PE36:1; PE38:6; PE40:7; PE40:5; PE 18:1-0:0; PE 18:2-0:0**; PE-O 40:6; **PI:18:0-0:0; PI:18:1-0:0; PI:20:4-0:0**; **PS36:1; PS40:5**; CE15:0; CE17:0; CE17:1; CE18:0; CE18:1; CE18:2; CE18:3; CE20:1; CE20:2; CE20:3; CE20:4; CE22:1; CE22:4; CE22:5; CE24:5
**X+/M+ (4.5)**	Dec: SM32:0; **PE36:5**; DG14:1-16:0
	Inc: Cer16:0; Cer22:0; Cer24:0; MHC18:0; **GM3 22:0; GM3 24:1**; LPC14:0; LPC16:1; LPC18:0; LPC20:0; LPC20:1; LPC24:0; PC29:0; PC32:0; **PC-O 32:0; PC-O 35:4; PC-O 36:5**; **PE34:3; PE34:1; PE36:3; PE36:2; PE 16:0-0:0; PE 18:1-0:0; PE 18:2-0:0**; **PI:18:1-0:0**; CE16:0
**X-/M+ (6)**	Dec: None decreased
	Inc: Cer16:0; Cer18:0; Cer20:0; Cer22:0; Cer24:0; Cer24:1; MHC18:0; SM36:3; SM38:1; SM39:1; SM41:2; PC:18:1-18:3; PC31:0; PC32:1; PC33:2; PC33:1; PC33:0; PC34:5; PC34:1; PC35:4; PC35:3; PC35:2; PC35:1; PC35:0; PC36:3; PC36:2; PC36:1; PC36:0; PC37:5; PC37:4; PC38:4; PC38:2; PC39:7; PC39:6; PC39:5; PC40:8; PC40:5; **PC-O 20:0-0:0**; **PE38:6; PE40:7; PE40:6; PE40:5**; PE-O 40:6; **PI:18:0-0:0; PI:18:1-0:0**; CE16:2; CE17:0
**X+/M- (6)**	Dec: None decreased
	Inc: Cer16:0; Cer20:0; Cer22:0; Cer24:0; Cer24:1; MHC16:0; MHC20:0; MHC22:0; MHC24:0; **DHC16:0; DHC22:0; DHC24:0**; **GM3 24:1**; SM34:1; SM35:2; SM37:2; SM38:1; SM38:2; SM39:1; SM41:1; SM41:1; SM41:2; SM42:1; LPC17:0; LPC18:0; PC:18:1-18:3; PC30:0; PC31:0; PC32:3; PC32:2; PC32:1; PC32:0; PC33:2; PC33:1; PC33:0; PC34:1; PC34:0; PC35:4; PC35:2; PC35:1; PC35:0; PC36:3; PC36:2; PC36:1; PC37:4; PC38:5; PC38:2; PC40:5; PC40:4; **PC-O 32:2; PC-O 32:1; PC-O 32:0; PC-O 34:2; PC-O 34:1; PC-O 36:5; PC-O 36:3; PC-O 40:7; PC-O 18:1-0:0; PC-O 20:0-0:0**; PC-P 36:2; **PE32:1; PE34:2; PE34:1; PE35:2; PE36:2; PE36:1; PE40:7; PE40:6; PE40:5; PE 16:0-0:0; PE 18:0-0:0; PE 18:1-0:0; PE 18:2-0:0; PE 22:6-0:0**; PE-O 40:6; **PI34:1; PI36:1; PI36:2; PI40:6; PI:18:1-0:0**; **PS36:1; PS38:3; PS38:4; PS40:5; PS40:6**; CE15:0; CE16:0; CE16:2; CE17:0; CE17:1; CE18:0; CE18:1; CE20:1; CE22:1; CE24:1; COH
**X+/M+ (6)**	Dec: PC37:6; PC37:5
	Inc: dhCer16:0; dhCer18:0; Cer16:0; Cer18:0; Cer20:0; Cer22:0; Cer24:0; Cer24:1; MHC16:0; MHC22:0; **DHC16:0; DHC22:0; DHC24:0; DHC24:1**; **GM3 16:0; GM3 22:0; GM3 24:1**; SM36:1; SM37:2; SM38:2; SM39:1; LPC14:0; LPC20:1; LPC22:5; PC:16:0-20:4; PC31:1; PC32:1; PC32:0; PC33:0; PC34:3; PC34:1; PC34:0; PC35:4; PC35:2; PC35:1; PC35:0; PC36:3; PC36:2; PC36:1; PC37:4; PC38:5; PC38:3; PC38:2; PC40:4; **PC-O 32:1; PC-O 32:0; PC-O 34:4; PC-O 34:3; PC-O 34:2; PC-O 34:1; PC-O 36:5; PC-O 36:4; PC-O 36:1; PC-O 38:5; PC-O 38:4; PC-O 20:0-0:0; PC-O 22:1-0:0**; PC-P 32:0; PC-P 34:3; PC-P 34:1; PC-P 36:2; PC-P 38:5; **PE32:1; PE34:3; PE34:2; PE34:1; PE35:2; PE35:1; PE36:3; PE36:2; PE36:1; PE38:6; PE38:4; PE38:3; PE40:7; PE40:6; PE40:5; PE40:4; PE 16:0-0:0; PE 18:0-0:0; PE 18:1-0:0; PE 18:2-0:0; PE 20:4-0:0**; PE-O 40:6; PE-P 16:0-22:6; PE-P 18:0-22:6; **PI34:1; PI36:3; PI36:4; PI38:5; PI38:6**; **PS38:3; PS40:5; PS40:6**; PG18:1-18:1; CE16:2; CE18:1; CE18:3; CE20:1; CE20:2; CE20:3; CE20:4; CE20:5; CE22:1; CE22:4; CE22:5; CE24:1; CE24:5; COH; **TG16:1-16:1-18:1**
**X-/M+ (7.5)**	Dec: SM34:0; **TG14:1-16:0-18:1; TG14:1-16:1-18:0; TG15:0-18:1-16:0; TG15:0-18:1-16:0**
	Inc: dhCer16:0; Cer16:0; Cer20:0; Cer22:0; Cer24:0; Cer24:1; MHC20:0; MHC22:0; SM41:2; LPC14:0; LPC15:0; LPC16:1; LPC17:0; LPC17:1; LPC18:1; LPC18:2; LPC18:3; LPC20:1; LPC20:2; LPC20:3; LPC20:4; PC:18:1-18:3; PC:16:0-22:6; PC29:0; PC30:0; PC31:1; PC31:0; PC32:1; PC32:0; PC33:2; PC33:1; PC33:0; PC34:1; PC35:4; PC35:3; PC35:1; PC36:3; PC36:2; PC36:1; PC37:4; PC38:2; PC39:7; PC39:6; PC39:5; PC40:8; PC40:7; PC40:6; PC40:5; PC40:4; **PC-O 34:3; PC-O 34:2; PC-O 34:1; PC-O 36:5; PC-O 40:7; PC-O 20:1-0:0**; PC-P 34:1; PC-P 38:6; PC-P 38:5; PC-P 40:6; **PE34:3; PE34:2; PE34:1; PE35:2; PE35:1; PE36:3; PE36:2; PE38:6; PE40:7; PE40:6; PE40:5; PE 16:0-0:0; PE 18:1-0:0; PE 18:2-0:0**; PE-O 40:6; PE-P 16:0-22:6; **PI34:1; PI36:3; PI36:4; PI38:4; PI38:5; PI38:6**; **PS38:4; PS40:5; PS40:6**; COH; DG14:0-18:1; DG16:1-18:1; DG16:0-18:1; DG18:0-16:1; DG18:1-18:2; DG18:1-18:1
**X+/M- (7.5)**	Dec: None decreased
	Inc: Cer16:0; Cer20:0; Cer22:0; Cer24:0; Cer24:1; MHC16:0; MHC22:0; MHC24:0; **DHC16:0; DHC22:0; DHC24:0; DHC24:1**; SM36:1; SM38:1; SM38:2; SM41:1; SM41:2; SM42:1; LPC14:0; LPC15:0; LPC16:0; LPC16:1; LPC17:0; LPC17:1; LPC18:0; LPC18:1; LPC18:2; LPC18:3; LPC20:0; LPC20:1; LPC20:2; LPC20:3; LPC20:4; LPC20:5; LPC22:5; LPC22:6; PC30:0; PC31:1; PC31:0; PC32:1; PC32:0; PC33:3; PC33:2; PC33:1; PC33:0; PC34:4; PC34:3; PC34:2; PC34:1; PC34:0; PC35:4; PC35:3; PC35:1; PC36:6; PC36:3; PC36:2; PC36:1; PC37:4; PC38:7; PC38:3; PC38:2; PC39:7; PC40:8; PC40:6; PC40:4; **PC-O 32:1; PC-O 32:0; PC-O 34:3; PC-O 34:2; PC-O 34:1; PC-O 40:7; PC-O 16:0-0:0; PC-O 18:0-0:0; PC-O 18:1-0:0; PC-O 20:1-0:0; PC-O 22:1-0:0**; PC-P 30:0; PC-P 34:3; PC-P 36:2; **PE32:1; PE34:3; PE34:2; PE34:1; PE35:2; PE35:1; PE36:3; PE36:2; PE36:1; PE38:6; PE40:7; PE40:6; PE40:5; PE 18:0-0:0; PE 18:1-0:0; PE 20:4-0:0; PE 22:6-0:0**; PE-O 40:6; PE-O 18:0-22:5; **PI34:1; PI36:2; PI36:3; PI36:4; PI38:5; PI38:6; PI40:5**; **PS40:5; PS40:6**; CE14:0; CE16:2; COH; DG14:0-18:1; DG16:0-18:2; DG16:1-18:1; DG16:0-18:1; DG18:0-16:1; DG18:2-18:2; DG18:1-18:2; DG18:1-18:1; DG18:1-20:4; DG18:1-20:3
**X+/M+ (7.5)**	Dec: **TG18:0-18:2-18:2; TG18:1-18:1-20:4**
	Inc: Cer20:0; Cer22:0; MHC18:0; MHC22:0; **DHC22:0**; **GM3 16:0; GM3 22:0; GM3 24:1**; SM41:2; LPC16:0; LPC16:1; LPC17:1; LPC18:0; LPC18:1; LPC18:2; LPC18:3; LPC20:0; LPC20:1; LPC20:2; LPC20:3; LPC20:4; LPC20:5; LPC22:5; LPC22:6; PC:18:1-18:3; PC29:0; PC33:0; PC35:4; PC35:2; PC35:0; PC36:3; PC36:0; **PC-O 32:0; PC-O 34:3; PC-O 16:0-0:0; PC-O 18:0-0:0; PC-O 18:1-0:0; PC-O 20:0-0:0; PC-O 22:1-0:0**; **PE32:1; PE34:2; PE36:3; PE36:2; PE 16:0-0:0; PE 18:0-0:0; PE 18:1-0:0; PE 18:2-0:0; PE 20:4-0:0; PE 22:6-0:0**; **PI34:1; PI36:1; PI36:3; PI:18:0-0:0**; PG18:1-18:1; CE17:0; CE18:1; CE22:0; CE22:4

Comprehensive listing of all lipid species with altered levels during HCC. Lipid species with the most decreased and increased levels are shown in bold text with underlining. Key: x−m−, non-transgenic control; x+m−, *xmrk* oncogene expression; x−m+, *Myc* oncogene expression; x+m+, *xmrk* and *Myc* oncogene expression; *Inc* increase, *Dec* decrease

The double transgenics also had unique lipid species characteristics. At 1.5 dpt, we found that x+m+ fish also had lipid species decreased in x−m+ samples and PC(O)s, additional PEs, PI 38:4 and PS 38.4, while unlike x+m−, x+m+ had increased PI 34.1 and decreased PC(O) 36:4 and PE 36:3 (Table 1). Double transgenics at 3 dpt exhibited similar lipid species regulation to x−m+ samples; however, x+m+ fish also had several TGs increased (Table 1). At 4.5 dpt, x+m+ lipid species levels in liver tissues resembled those in x+m− samples, but not to the same extent, and expressed increased GM3 (22:0 and 24:1) (Table 1). At 6 dpt, x+m+ liver profiles were similar to those observed in x+m−, but x+m+ samples had increased levels of several TGs (Table 1). Data from 7.5 dpt time points showed that double transgenics exhibited similar PC(O), PE, and PI lipid species upregulation as observed in the single transgenics (Table 1). However, x+m+ fish did not have significant levels of PS species, but only they expressed GM3 species (Table 1).

### Expression of lipid metabolism genes in liver tumors

**Fig. 5:**
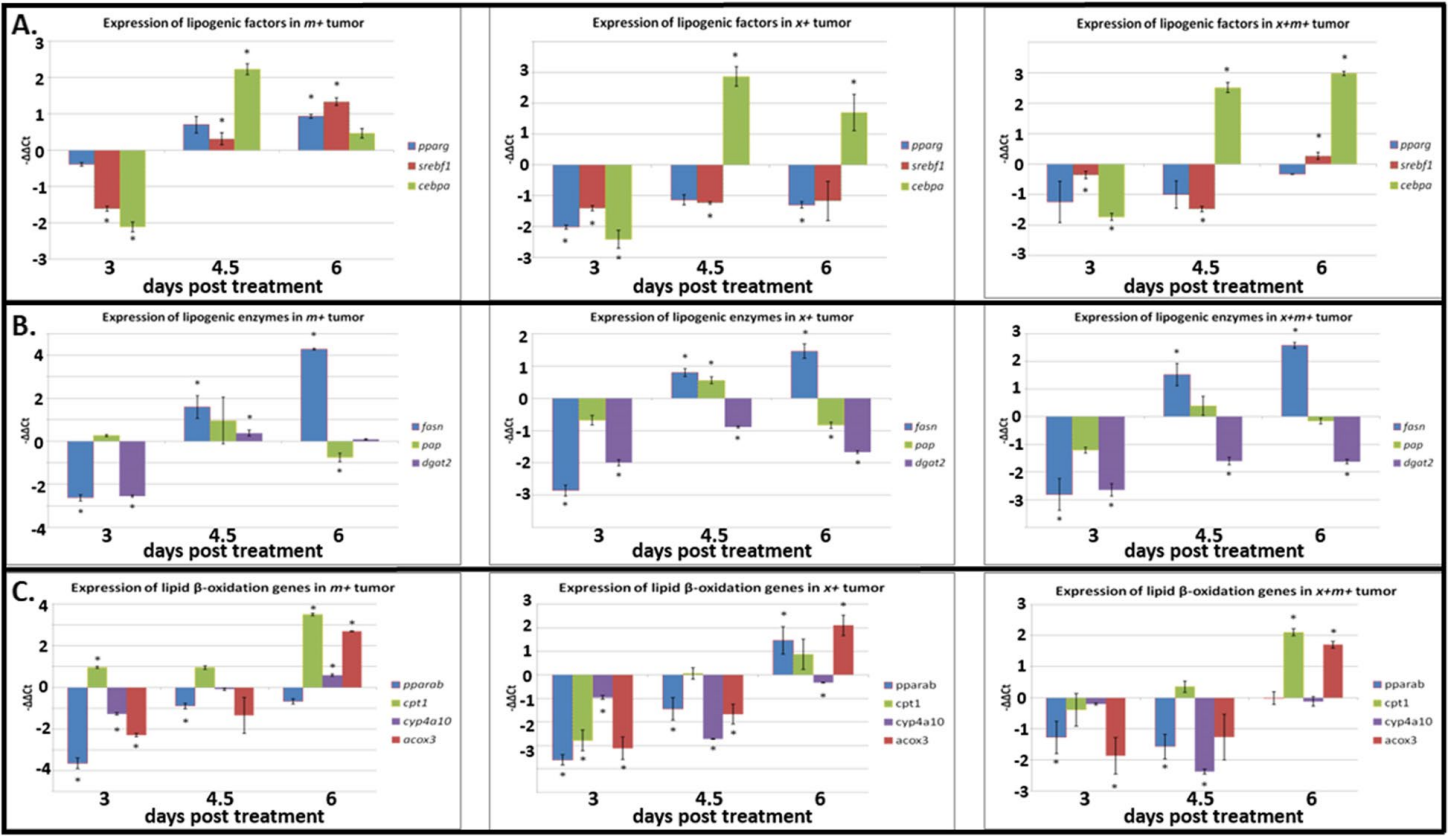
Expression profiles for lipid metabolism genes. The expression of lipogenic factors, enzymes, and lipid β-oxidation genes is altered in x−m+, x+m−, and x+m+ tumors at 3, 4.5, and 6 dpt. **A** Lipogenic factors expression profiles. **B** Lipogenic enzyme expression profiles. **C** Lipid β-oxidation gene expression profiles (see text for gene definitions). *N* = 3; *p < 0.05

We next measured the expression of key metabolic genes in DOX-treated liver tissue specimens at selected stages (3, 4.5, and 6 dpt) corresponding to the rapid period of transformation into hyperplasia and HCC (Fig. 1B–D). These genes included: lipogenic factors (peroxisome proliferator-activated receptor gamma [*pparg*], sterol regulatory element-binding transcription factor 1 [*srebf1*], CCAAT/enhancer-binding protein alpha [*cebpa*]), lipogenic enzymes (fatty acid synthase [*fasn*], phosphatidic acid phosphatase [*pap*], diacylglycerol O-acyltransferase 2 [*dgat2*]), and lipid β-oxidation genes (peroxisome proliferator-activated receptor alpha b [*pparab*], carnitine palmitoyltransferase 1 [*cpt1*], cytochrome P450 monooxygenase [*cyp4a10*], acyl-coenzyme A oxidase 3 [*acox3*] )[23–32]. The majority of these genes exhibited downregulation at 3 dpt compared to the controls, with the exception of *cpt1*, which was upregulated in x−m+ samples (Fig. 5A, B). At 4.5 dpt, in x−m+ samples, *cebpa*, *dgat2*, *fasn*, and *srebf1* were upregulated and *pparb* was downregulated, in x+m− samples, *dgat2* and *srebf1* were downregulated, while *acox3*, *cebpa*, *cyp4a10*, *fasn*, *pap*, and *pparab* showed increased expression, and in x+m+, *cyp4a10*, *dgat2*, *pparab*, and *srebf1* expression was decreased, while *cebpa* and *fasn* were upregulated (Fig. 5A, B). At 6 dpt, x−m+ transgenics showed upregulated *acox3*, *cpt1*, *cyp4a10*, *fasn*, *pparg*, and *srebf1* expression, and decreased *pap* expression; x+m− showed downregulated *dgat2*, *pap*, and *pparg* expression, and increased *acox3*, *cebpa*, *fasn*, and *pparab* expression, while x+m+ samples exhibited upregulation of *acox3*, *cebpa*, *cpt1*, *fasn*, and *srebf1*, and downregulation of *cyp4a10* and *dgat2* expression with *pap* and *pparg* expression being unchanged (Fig. 5A, B).

### Association of lipid class regulation with HCC disease progression

**Fig. 6:**
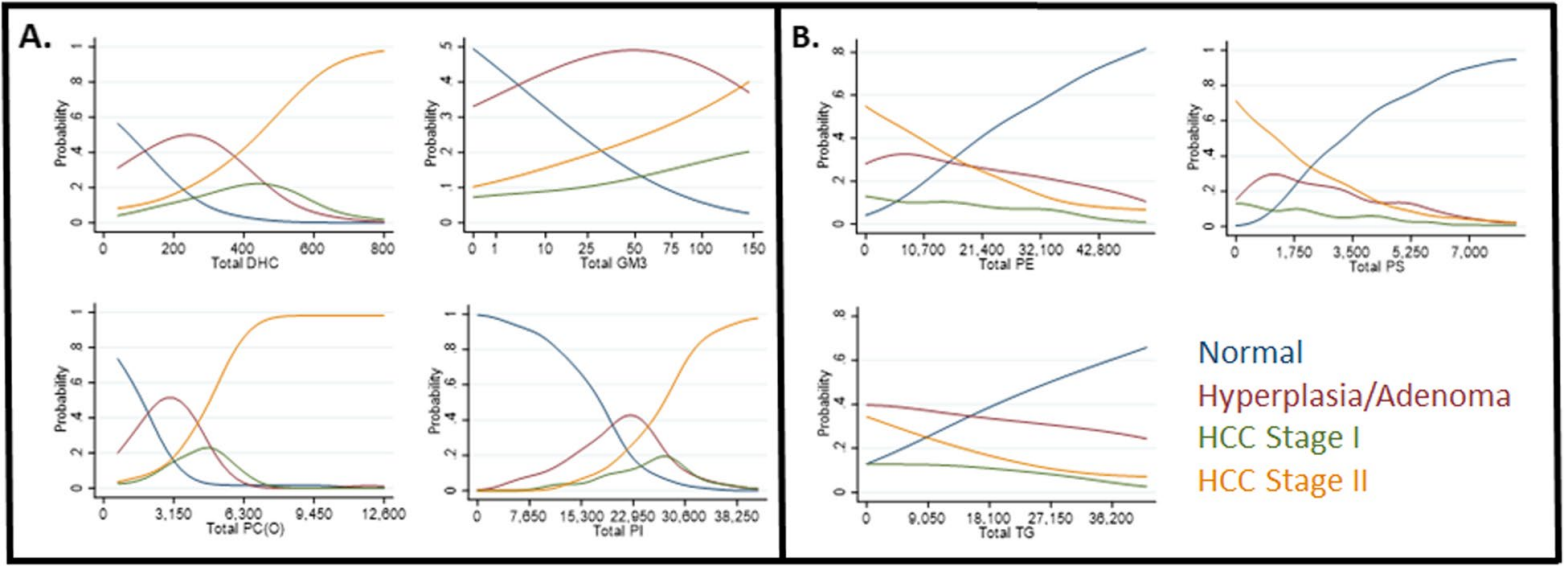
Total lipid class level association with HCC disease stage. Univariate modeling shows the probability that individual total lipid class levels are associated with normal or disease stage phenotypes. Plots show total lipid levels versus probability of disease progression. **A** DHC, GM3, PC(O), and PI plot. **B** PE, PS, and TG plot. *X*-axis units: pmol/mg. Key: “OR” (odds ratio); dark blue lines (normal); dark red lines (hyperplasia/ adenoma); green lines (HCC stage I); orange lines (HCC stage II)

To identify lipid classes that may contribute to HCC disease progression, we related each lipid class to HCC disease stage using univariate ordinal logistic regression models. Univariate analysis showed that total DHC, GM3, PC(O), and PI levels were correlated with increased promotion of HCC II (Fig. 6A), while increasing total PS, PE, and TG levels were associated with the normal, non-HCC phenotype (Fig. 6B, see supplementary Figs. 3, 4, 5 and 6 for additional univariate data sets). However, since univariate analyses of individual lipid classes may not accurately reflect their potential systemic role in HCC progression, we also used multivariate fractional polynomial (MFP) analysis to interrelate the potential effect of each lipid class on HCC development (Table 2). The MFP analysis confirmed that DHC, PC(O), GM(3), PS, PE, PI, and TG levels were associated with the progression of HCC during oncogenic induction (Table 2, see supplementary Fig. 1 for excluded lipid class data sets). Specifically, four lipid sub-classes displayed increased odds of disease severity: DHC [OR = 2.17; (1.30–3.61); *p* = 0.003], GM3 [OR = 1.64; (1.28–2.11); *p* < 0.001], PC(O) [OR = 4.32; (1.87–10.0); *p* = 0.001], and PI [OR = 3.22; (1.85–5.62); *p* < 0.001] had increased odds of disease severity per 40, 10 square root, 630, and 1530 units, respectively (Table 2). The opposite relationship was observed for 3 lipid sub-classes: PE [OR = 0.52; (0.29–0.95); *p* = 0.033], PS [OR = 0.29; (0.14–0.61); *p* = 0.001], and TG [OR = 0.73; (0.60–0.89); *p* = 0.001] that had decreased odds of disease severity per 2140, 350, and 1810 units, respectively (Table 2). The MFP analysis produced scaling factors of 40 (DHC), 10 (GM3), 630 (PC(O)), 2140 (PE), 1530 (PI), 350 (PS), and 1810 (TG) units, respectively. Also, the final model had the most superior Akaike information criterion [33] values (88.70) and *R*^2^ (0.55) among examined models.

**Table 2:** Multivariate fractional polynomial analysis of lipid class associations with disease stage

Lipid class	Odds ratio	Outcome
**DHC**	2.17 (1.30–3.61); *p* = 0.003	117% increase in the odds of moving to a worse cancer stage
**GM3**	1.64 (1.28–2.11); *p* < 0.001	64% increase in the odds of moving to a worse cancer stage
**PC(O)**	4.32 (1.87–10.0); *p* = 0.001	332% increase in the odds of moving to a worse cancer stage
**PI**	3.22 (1.85–5.62); *p* < 0.001	222% increase in the odds of moving to a worse cancer stage
**PE**	0.52 (0.29–0.95); *p* = 0.033	48% decrease in the odds of moving to a worse cancer stage
**PS**	0.29 (0.14–0.61); *p* = 0.001	71% decrease in the odds of moving to a worse cancer stage
**TG**	0.73 (0.60–0.89); *p* = 0.001	27% decrease in the odds of moving to a worse cancer stage

The results of the univariate ordinal logistic regression models were placed into a multivariate fractional polynomial analysis with backward selection variable procedures enabled to determine which lipid classes significantly modulated the HCC disease stage. All results were linear except that total GM3 used a square root nonlinear transformation (AIC =88.7 and *R*^2^ = 0.55). *OR* odds ratio; *p* < 0.05

The results of the univariate ordinal logistic regression models were placed into a multivariate fractional polynomial analysis with backward selection variable procedures enabled to determine which lipid classes significantly modulated the HCC disease stage. All results were linear except that total GM3 used a square root nonlinear transformation (AIC =88.7 and *R*^2^ = 0.55). OR, odds ratio; *p* < 0.05.

## Discussion

Lipids and their metabolites can play roles as energy, signaling, and/or prognostic biomarkers in HCC [34, 35], but oncogene-mediated regulation of specific lipids and related metabolomic genes during liver cancer progression have not been well studied. Therefore, we utilized transgenic zebrafish models of HCC expressing the liver cancer–inducing oncogenes, *xmrk* and *Myc* [8–11] to analyze lipidomic profiles and lipid metabolic genes at different HCC stages. *Xmrk* is a fish homolog of the human epidermal growth factor receptor (EGFR), a transmembrane receptor tyrosine kinase that regulates multiple signaling pathways controlling cell proliferation, migration, and inhibition [36]. EGFR plays a complex role in liver cancer where it is typically upregulated [37]. Some forms of *Myc* act as transcription factors that can modulate EGFR gene expression [38], and *Myc* amplification is correlated with HCC development and lipogenesis [39, 40]. Further, EGFR stimulation can upregulate *Myc* expression in HCC [41]. We found that x+m−, x−m+, and x+m+ liver samples exhibited characteristics of HCC oncogenesis [6] within 7.5 dpt (Fig. 1) indicating that these oncogenes modulate phenotypic attributes of HCC. These data suggest that combined transgene induction may enhance disease progression perhaps via *xmrk*-mediated self-potentiation by a signaling loop integrating *Myc*.

The presence of increased lipid levels at later time points in the single and double transgenics (Fig. 2) suggests that *xmrk* and *Myc* might promote HCC oncogenesis via specific types of lipids, but surprisingly, few of the lipid classes that we detected were altered (Fig. 3, supplementary Fig. 1). During HCC, lipid metabolites and lipid-related gene regulation can exhibit extensive up- and downregulation [42], and we observed altered regulation of some lipid classes, e.g., DHC, PC(O), GM3, PI, PE, and PS (Fig. 3A, B), where a general upward trend in levels was found beginning at 4.5 or 6 dpt, and TGs, where there is a general downward trend across all time points (Fig. 3B). The lipid classes PE and PI also can be dysregulated and have been correlated with HCC disease and its progression [43, 44]. Further, the overall increasing relative levels of lipid classes across the time points (Fig. 4) may suggest ongoing lipid metabolic reprogramming which is observed in human HCC as well, where it has been associated with enhanced lipid synthesis [45]. In summary, altered lipid levels could represent a response to the different metabolic and/or signaling demands encountered during HCC progression.

Analysis of specific lipid species showed that induction of *xmrk* and *Myc* alone in HCC may regulate largely distinct sets of lipid species suggesting that each transgene may target lipid-based metabolism and signaling mechanisms (Table 1). In human HCC cell lines, the expression of EGFR and other genes has been correlated with lipid species regulation [46]. Further, the *Myc* oncogene can modulate some lipid species during HCC, particularly members of the PG class [40]. Recently, EGFR and *Myc* signals have been shown to be integrated into a signaling axis that modulates oncogenesis [41]. Therefore, expressing both oncogenes simultaneously may result in the utilization of different lipid species when compared to the single transgene models. Interestingly, when we compared the lipid species associated with the single transgenics with lipid species associated with the double transgenics at the same time points, we found that at 1.5 and 3 dpt, *Myc* appeared to drive lipid species levels in the double transgenics, but that from 4.5 to 6 dpt, *xmrk* may supplant this role in x+m+ HCC liver samples (Table 1). These results suggest that shifts in the metabolic or signaling demands associated with particular HCC disease stages may be responsible for the transposition of *xmrk* and *Myc* regulation of lipid species in x+m+. However, neither oncogene has a primary role in modulating lipid species regulation in x+m+ samples at 7.5 dpt, when mortality begins to appear (Table 1). Lipid-based mechanisms can regulate cell death mechanisms in cancer [47], and some lipid classes, e.g., GM3, which exhibits increased levels in late HCC stage x+m+ samples (Fig. 3A), can inhibit EGFR function [35] suggesting that the levels of some lipids in the transgenic models could be associated with late HCC stage cell death.

Distinct x*mrk* and *Myc* temporal regulation of lipids could mean that these oncogenes modulate different lipid metabolic genes. Measuring the expression profiles of these genes over various time points allows assessment of their status as biomarkers without necessitating the analysis of the corresponding enzyme profiles or their mechanistic role in regulating lipid levels. The expression profiles for the lipogenic factors, *pparg* and *srebf1*, and for the enzyme, *dgat2*, in x+m+ samples are generally similar to those observed in *xmrk* only model (Fig. 5A), suggesting that *xmrk* may control these genes; although, *Myc* may be counteracting *xmrk* regulation of *srebf1* by 6 dpt (Fig. 5A). Activation of *pparg* causes growth inhibition and cell death in HCC cells [23] suggesting that *xmrk*-mediated reduction of *pparg* expression (Fig. 5A) may promote oncogenesis in HCC. Increased expression of *srebf1* is associated with cell proliferation in liver cancer [28] indicating that *Myc* may contribute to HCC oncogenesis at 6 dpt (Fig. 5A). As suppression of the lipogenic enzyme gene, *dgat2*, is associated with increased HCC cell proliferation [30], *xmrk* induction also could be driving disease progression by targeting *dgat2* function (Fig. 5B). Expression of the lipid β-oxidation genes exhibited more complex regulation with *pparab* correlated with *Myc*, *cpt1* with both *xmrk* and *Myc*, and *cyp4a10* with *xmrk* (Fig. 5C). As reduced expression of *pparab* is associated with increased HCC development [27], *Myc* could be promoting liver cancer onset by suppressing lipid breakdown via *pparab* function. Further, as increased *cpt1* is associated with HCC progression [31], these data support the suggestion that *xmrk* and *Myc* induction together promote the development of liver cancer via enhanced *cpt1* expression. Interestingly, *cyp4a10* expression is increased in liver tumor development in mice [26], but we found that *cyp4a10* expression in the x+m+ samples was reduced suggesting that *xmrk* may counteract HCC progression through this gene (Fig. 5C). Therefore, *xmrk* and *Myc* may modulate distinct subsets of lipid metabolic genes that may have some utility as biomarkers of HCC progression.

In support of this concept, altered lipid levels may act as an indicator of HCC disease progression as lipid levels change during HCC [42, 48]. DHC is associated with the regulation of differentiation, proliferation, and programmed cell death [49], and its elevated levels (Fig. 6A, Table 2) may be an indicator of oncogenic progression. GM3 can suppress cancer progression [50]; therefore, its elevation during HCC progression (Fig. 6A, Table 2) could be a biomarker of a cellular response to counteract oncogenesis. PC(O) is a phospholipid, and some phospholipid metabolites can mediate proliferative growth and programmed cell death in cancer [51]; therefore, PC(O) elevation (Fig. 6A, Table 2) may be a marker of advanced HCC disease stage. PI species can participate in cell signaling in cancer [52] suggesting that increased PI levels during HCC (Fig. 6A, Table 2) could indicate either pro- or antioncogenic responses. PE phospholipids exhibit increased levels during cancer where they play a role in cell division and death [53]. Increased PE (Fig. 6B, Table 2) during HCC progression could represent activation of a cell death mechanism that counteracts liver cancer. PS phospholipid levels may regulate phagocytosis, immunosuppression, tumor growth, and metastasis [54]. Therefore, increased PS levels (Fig. 6B, Table 2) could indicate phagocytic anticancer activity. TG levels in the liver are regulated by uptake, secretory, and metabolic mechanisms [55], and higher TG levels (Fig. 6B, Table 2) could represent reduced mobilization of TG for driving mechanisms that promote HCC oncogenesis. Thus, altered levels of some lipid classes and their constituent species may act as diagnostic biomarkers of HCC disease progression.

When performing liver lipid analysis in HCC, it is important to bear in mind that changes in the diet can affect liver lipid content [56]. For this reason, our control and DOX-exposed fish were fed the same diet and amount to rule out inducing any effect from qualitative or quantitative variations in food intake. Moreover, in rats, dietary change can affect liver lipid content in non-alcoholic fatty liver disease (NAFLD) models, but the main lipid classes that displayed a strong variability in the liver were TGs and the lysophospholipids, LPC and LPE, which were higher in high-fat diet (HFD) rat livers [57]. This observation is consistent with our data as neither LPC nor LPE were identified as biomarkers of cancer progression (Figure 6). Further, unlike in many cases of HFD, where TGs levels are dramatically increased in the liver [57–60], in the data we derived, decreased TG levels indicate a non-cancerous liver (Figure 6B). Therefore, the lipid species identified in this study are not responding to diet variations and, instead, are hepatocyte cancer state specific. As zebrafish are lecithotrophic organisms, i.e., the embryo receives no nutrition other than what the yolk sac contains originally, a difference in the lipid content deposited in the yolk sac by the mother during oocyte formation might affect the future lipid content of the liver in adults. However, this effect is unlikely to have occurred in this study, because reproductive females were from similar batches of fish and were all fed the same quality and quantity of food, so a variation in yolk sac lipid content should not be present or responsible for the lipid variation observed in the HCC fish models.

## Conclusions

These studies indicate that induction of *xmrk*, *Myc*, and *xmrk*/*Myc* causes temporal alteration of lipid class levels and metabolic gene expression at different stages of HCC. Increased lipid class levels are associated with HCC progression or restoration of the normal phenotype and may act as predictive biomarkers of HCC.

## Supplementary Information

**Additional file 1: Supplementary Figure 1.** Expression profiles for lipid classes not associated with HCC disease progression. Additional lipid classes were detected in HCC zebrafish control and transgenic livers over the 1.5 to 7.5 dpt time course but were not associated with HCC disease progression by the MFP analysis. A. dhCer, B. Cer, C. MHC, D. THC, E. SM, F. LPAF, G. LPC, H. PC, I. PC(P), J. PE(O), K. PE(P), L. LPE, M. LPI, N. PG, O. CE, P. COH, Q. DG. Color key: x-m-, brown column; x-m+, blue column; x+m-, red column; x+m+, green column. “*” = *p* < 0.05; “**” = *p* < 0.01

**Additional file 2: Supplementary Figure 2.** Expression profiles for lipid species after transgene induction. A-C. Total number of lipid species showing a decrease (dark gray) or an increase (light gray) in all transgene types from 1.5 to 7.5 dpt. D. Heat map showing all lipid species detected at 7.5 dpt in all transgene types compared to DOX only (no transgene) control liver tissues. A red band shows an increase of a particular lipid species in cancer cells, a green band shows a decrease of a particular lipid species, and a dark band indicated no change. Data expressed as log2 transformation of the value of lipid species/value of control lipid species.

**Additional file 3: Supplementary Figure 3.** Univariate modelling analysis for dhCer, Cer, MHC, THC and SM lipid classes. Several lipid classes analyzed with univariate modelling analysis were not associated with HCC disease progression by MFP analysis. A. dhCer, B. Cer, C. MHC, D. THC, E. SM. X-axis units: pmol/mg. Key: “OR” (odds ratio); dark blue lines (normal); dark red lines (hyperplasia/ adenoma); green lines (HCC stage I); orange lines (HCC stage II).

**Additional file 4: Supplementary Figure 4.** Univariate modelling analysis for DG, PC(P), LPAF, LPC and PC lipid classes. Several lipid classes analyzed with univariate modelling analysis were not associated with HCC disease progression by MFP analysis. A. DG, B. PC(P), C. LPAF, D. LPC, E. PC. X-axis units: pmol/mg. Key: “OR” (odds ratio); dark blue lines (normal); dark red lines (hyperplasia/ adenoma); green lines (HCC stage I); orange lines (HCC stage II).

**Additional file 5: Supplementary Figure 5.** Univariate modelling analysis for LPE, PE(P), LPI, PE(O) and PG lipid classes. Several lipid classes analyzed with univariate modelling analysis were not associated with HCC disease progression by MFP analysis. A. LPE, B. PE(P), C. LPI, D. PE(O), E. PG. X-axis units: pmol/mg. Key: “OR” (odds ratio); dark blue lines (normal); dark red lines (hyperplasia/ adenoma); green lines (HCC stage I); orange lines (HCC stage II).

**Additional file 6: Supplementary Figure 6.** Univariate modelling analysis for CE and COH lipid classes. Some lipid classes analyzed with univariate modelling analysis were not associated with HCC disease progression by MFP analysis. A. CE, B. COH. X-axis units: pmol/mg. Key: “OR” (odds ratio); dark blue lines (normal); dark red lines (hyperplasia/ adenoma); green lines (HCC stage I); orange lines (HCC stage II).

**Additional file 7: Supplementary Table 1.** List of qPCR primers.

## Data Availability

The datasets used and/or analyzed during the current study are available from the corresponding author on reasonable request.

## References

[1] Santos CR, Schulze A (2012). Lipid metabolism in cancer. FEBS J..

[2] Bian X, Liu R, Meng Y, Xing D, Xu D, Lu Z (2020). Lipid metabolism and cancer. J Exp Med..

[3] Hu B, Lin JZ, Yang XB, Sang XT (2020). Aberrant lipid metabolism in hepatocellular carcinoma cells as well as immune microenvironment: a review. Cell Prolif..

[4] Hason M, Bartůněk P (2019). Zebrafish models of cancer-new insights on modeling human cancer in a non-mammalian vertebrate. Genes (Basel)..

[5] Gamble JT, Elson DJ, Greenwood JA, Tanguay RL, Kolluri SK (2021). The zebrafish xenograft models for investigating cancer and cancer therapeutics. Biology (Basel)..

[6] Lam SH, Wu YL, Vega VB, Miller LD, Spitsbergen J, Tong Y (2006). Conservation of gene expression signatures between zebrafish and human liver tumors and tumor progression. Nat Biotechnol..

[7] Lu JW, Hsia Y, Tu HC, Hsiao YC, Yang WY, Wang HD (2011). Liver development and cancer formation in zebrafish. Birth Defects Res C Embryo Today..

[8] Li Z, Huang X, Zhan H, Zeng Z, Li C, Spitsbergen JM (2012). Inducible and repressable oncogene-addicted hepatocellular carcinoma in Tet-on xmrk transgenic zebrafish. J Hepatol..

[9] Li Z, Luo H, Li C, Huo X, Yan C, Huang X (2014). Transcriptomic analysis of a transgenic zebrafish hepatocellular carcinoma model reveals a prominent role of immune responses in tumour progression and regression. Int J Cancer..

[10] Zheng W, Li Z, Nguyen AT, Li C, Emelyanov A, Gong Z (2014). Xmrk, kras and myc transgenic zebrafish liver cancer models share molecular signatures with subsets of human hepatocellular carcinoma. PLoS One..

[11] Li Z, Zheng W, Li H, Li C, Gong Z (2015). Synergistic induction of potential Warburg effect in zebrafish hepatocellular carcinoma by co-transgenic expression of Myc and xmrk oncogenes. PLoS One..

[12] Li H, Lu JW, Huo X, Li Y, Li Z, Gong Z (2019). Effects of sex hormones on liver tumor progression and regression in Myc/xmrk double oncogene transgenic zebrafish. Gen Comp Endocrinol..

[13] Nakayama J, Makinoshima H (2020). Zebrafish-based screening models for the identification of anti-metastatic drugs. Molecules..

[14] Zhang X, Li C, Gong Z (2014). Development of a convenient in vivo hepatotoxin assay using a transgenic zebrafish line with liver-specific DsRed expression. PLoS One..

[15] Pei K, Gui T, Kan D, Feng H, Jin Y, Yang Y (2020). An overview of lipid metabolism and nonalcoholic fatty liver disease. Biomed Res Int..

[16] Puri P, Baillie RA, Wiest MM, Mirshahi F, Choudhury J, Cheung O (2007). A lipidomic analysis of nonalcoholic fatty liver disease. Hepatology..

[17] Westerfield M (2007). The Zebrafish Book. A guide for the laboratory use of zebrafish (*Danio rerio*). 5th Edition.

[18] Li C, Li P, Tan YM, Lam SH, Chan EC, Gong Z (2016). Metabolomic characterizations of liver injury caused by acute arsenic toxicity in zebrafish. PLoS One..

[19] Weir JM, Wong G, Barlow CK, Greeve MA, Kowalczyk A, Almasy L (2013). Plasma lipid profiling in a large population-based cohort. J Lipid Res..

[20] Fraher D, Sanigorski A, Mellett NA, Meikle PJ, Sinclair AJ, Gibert Y (2016). Zebrafish embryonic lipidomic analysis reveals that the yolk cell is metabolically active in processing lipid. Cell Rep..

[21] Sauerbrei W, Royston P (1999). Building multivariable prognostic and diagnostic models: transformation of the predictors by using fractional polynomials. J R Statist Soc A..

[22] Zhang Z (2016). Multivariable fractional polynomial method for regression model. Ann Transl Med..

[23] Hsu HT, Chi CW (2014). Emerging role of the peroxisome proliferator-activated receptor-gamma in hepatocellular carcinoma. J Hepatocell Carcinoma..

[24] Lu GD, Ang YH, Zhou J, Tamilarasi J, Yan B, Lim YC (2015). CCAAT/enhancer binding protein α predicts poorer prognosis and prevents energy starvation-induced cell death in hepatocellular carcinoma. Hepatology..

[25] Qu Q, Zeng F, Liu X, Wang QJ, Deng F (2016). Fatty acid oxidation and carnitine palmitoyltransferase I: emerging therapeutic targets in cancer. Cell Death Dis..

[26] Kuwata K, Inoue K, Ichimura R, Takahashi M, Kodama Y, Yoshida M (2016). Constitutive active/androstane receptor, peroxisome proliferator-activated receptor α, and cytotoxicity are involved in oxadiazon-induced liver tumor development in mice. Food Chem Toxicol..

[27] Xiao YB, Cai SH, Liu LL, Yang X, Yun JP (2018). Decreased expression of peroxisome proliferator-activated receptor alpha indicates unfavorable outcomes in hepatocellular carcinoma. Cancer Manag Res..

[28] Zhao X, Zhao L, Yang H, Li J, Min X, Yang F (2018). Pyruvate kinase M2 interacts with nuclear sterol regulatory element-binding protein 1a and thereby activates lipogenesis and cell proliferation in hepatocellular carcinoma. J Biol Chem..

[29] Che L, Paliogiannis P, Cigliano A, Pilo MG, Chen X, Calvisi DF (2019). Pathogenetic, prognostic, and therapeutic role of fatty acid synthase in human hepatocellular carcinoma. Front Oncol..

[30] Li Y, Li T, Jin Y, Shen J (2019). Dgat2 reduces hepatocellular carcinoma malignancy via downregulation of cell cycle-related gene expression. Biomed Pharmacother..

[31] Xu A, Wang B, Fu J, Qin W, Yu T, Yang Z (2019). Diet-induced hepatic steatosis activates Ras to promote hepatocarcinogenesis via CPT1α. Cancer Lett..

[32] Brohée L, Crémer J, Colige A, Deroanne C (2021). Lipin-1, a versatile regulator of lipid homeostasis, is a potential target for fighting cancer. Int J Mol Sci..

[33] Portet S (2020). A primer on model selection using the Akaike Information Criterion. Infect Dis Model..

[34] Hayes CN, Zhang P, Chayama K, Tirnitz-Parker JEE (2019). The role of lipids in hepatocellular carcinoma. Hepatocellular carcinoma.

[35] Sasaki N, Toyoda M, Ishiwata T (2021). Gangliosides as signaling regulators in cancer. Int J Mol Sci..

[36] Monroe JD, Basheer F, Gibert Y (2021). Xmrks the spot: fish models for investigating epidermal growth factor receptor signaling in cancer research. Cells..

[37] Komposch K, Sibilia M (2015). EGFR signaling in liver diseases. Int J Mol Sci..

[38] Perini G, Diolaiti D, Porro A, Della VG (2005). In vivo transcriptional regulation of N-Myc target genes is controlled by E-box methylation. Proc Natl Acad Sci U S A..

[39] Takahashi Y, Kawate S, Watanabe M, Fukushima J, Mori S, Fukusato T (2007). Amplification of c-myc and cyclin D1 genes in primary and metastatic carcinomas of the liver. Pathol Int..

[40] Gouw AM, Margulis K, Liu NS, Raman SJ, Mancuso A, Toal GG (2019). The MYC oncogene cooperates with sterol-regulated element-binding protein to regulate lipogenesis essential for neoplastic growth. Cell Metab..

[41] Pan Z, Liu C, Zhi Y, Xie Z, Wu L, Jiang M (2021). LIMK1 nuclear translocation promotes hepatocellular carcinoma progression by increasing p-ERK nuclear shuttling and by activating c-Myc signalling upon EGF stimulation. Oncogene..

[42] Budhu A, Roessler S, Zhao X, Yu Z, Forgues M, Ji J (2013). Integrated metabolite and gene expression profiles identify lipid biomarkers associated with progression of hepatocellular carcinoma and patient outcomes. Gastroenterology..

[43] Li Z, Guan M, Lin Y, Cui X, Zhang Y, Zhao Z (2017). Aberrant lipid metabolism in hepatocellular carcinoma revealed by liver lipidomics. Int J Mol Sci..

[44] Ismail IT, Elfert A, Helal M, Salama I, El-Said H, Fiehn O (2020). Remodeling lipids in the transition from chronic liver disease to hepatocellular carcinoma. Cancers (Basel)..

[45] Nakagawa H, Hayata Y, Kawamura S, Yamada T, Fujiwara N, Koike K (2018). Lipid metabolic reprogramming in hepatocellular carcinoma. Cancers (Basel)..

[46] Rivas Serna IM, Romito I, Maugeri A, Lo Re O, Giallongo S, Mazzoccoli G (2020). A lipidomic signature complements stemness features acquisition in liver cancer cells. Int J Mol Sci..

[47] Huang C, Freter C (2015). Lipid metabolism, apoptosis and cancer therapy. Int J Mol Sci..

[48] Sangineto M, Villani R, Cavallone F, Romano A, Loizzi D, Serviddio G (2020). Lipid metabolism in development and progression of hepatocellular carcinoma. Cancers (Basel)..

[49] Ogretmen B (2018). Sphingolipid metabolism in cancer signalling and therapy. Nat Rev Cancer..

[50] Zheng C, Terreni M, Sollogoub M, Zhang Y (2019). Ganglioside GM3 and its role in cancer. Curr Med Chem..

[51] Ridgway ND (2013). The role of phosphatidylcholine and choline metabolites to cell proliferation and survival. Crit Rev Biochem Mol Biol..

[52] Beloribi-Djefaflia S, Vasseur S, Guillaumond F (2014). Lipid metabolic reprogramming in cancer cells. Oncoscience..

[53] Tan LT, Chan KG, Pusparajah P, Lee WL, Chuah LH, Khan TM, Lee LH (2017). Targeting membrane lipid a potential cancer cure?. Front Pharmacol..

[54] Chang W, Fa H, Xiao D, Wang J (2020). Targeting phosphatidylserine for cancer therapy: prospects and challenges. Theranostics..

[55] Alves-Bezerra M, Cohen DE (2017). Triglyceride metabolism in the liver. Compr Physiol..

[56] Hodson L, Rosqvist F, Parry SA (2020). The influence of dietary fatty acids on liver fat content and metabolism. Proc Nutr Soc..

[57] Yaligar J, Gopalan V, Kiat OW, Sugii S, Shui G, Lam BD, Henry CJ (2014). Evaluation of dietary effects on hepatic lipids in high fat and placebo diet fed rats by in vivo MRS and LC-MS techniques. PLoS One..

[58] Handayani D, Meyer BJ, Chen J, Brown SH, Mitchell TW, Huang XF (2014). A high-dose Shiitake mushroom increases hepatic accumulation of triacylglycerol in rats fed a high-fat diet: underlying mechanism. Nutrients..

[59] Siersbæk M, Varticovski L, Yang S, Baek S, Nielsen R, Mandrup S, Hager GL (2017). High fat diet-induced changes of mouse hepatic transcription and enhancer activity can be reversed by subsequent weight loss. Sci Rep..

[60] Li X, Wang Z, Klaunig JE (2019). The effects of perfluorooctanoate on high fat diet induced non-alcoholic fatty liver disease in mice. Toxicology..

