# Supplementary figures and images for: Identification of novel lipid biomarkers in xmrk- and Myc-induced models of hepatocellular carcinoma in zebrafish
Source: Cancer Metab. 2022 Apr 4;10:7. doi: 10.1186/s40170-022-00283-y (PMC8981695; doi:10.1186/s40170-022-00283-y)

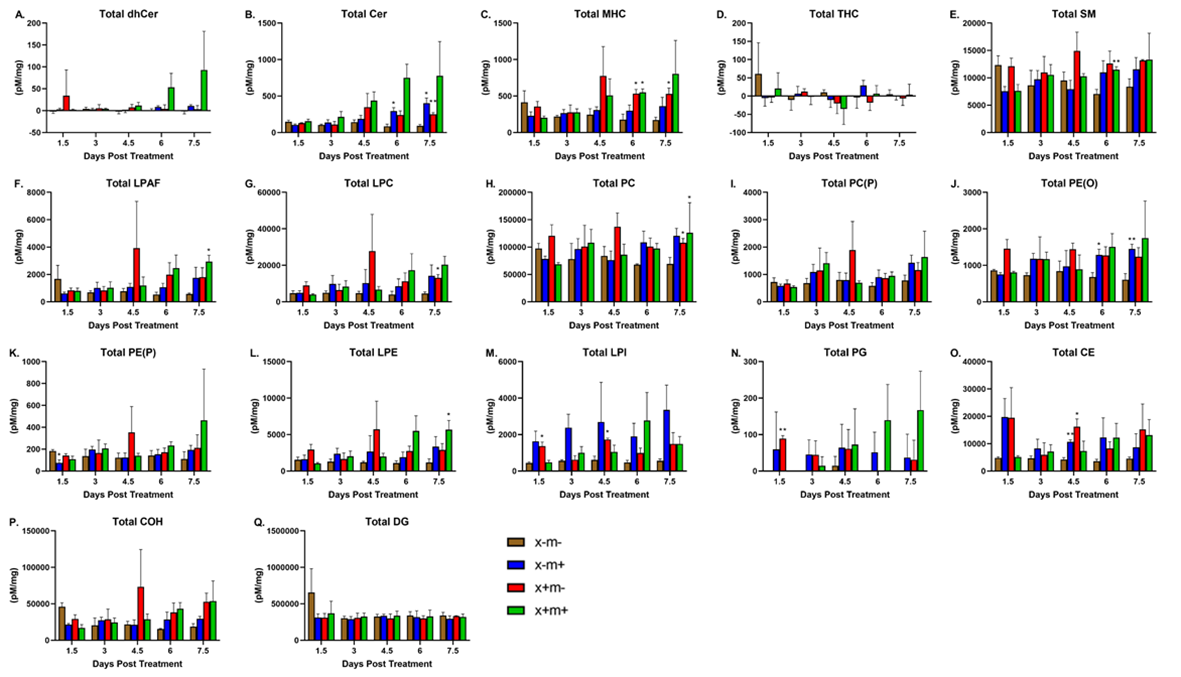

Supplement: Supplementary file 1 — Additional file 1: Supplementary Figure 1. Expression profiles for lipid classes not associated with HCC disease progression. Additional lipid classes were detected in HCC zebrafish control and transgenic livers over the 1.5 to 7.5 dpt time course but were not associated with HCC disease progression by the MFP analysis. A. dhCer, B. Cer, C. MHC, D. THC, E. SM, F. LPAF, G. LPC, H. PC, I. PC(P), J. PE(O), K. PE(P), L. LPE, M. LPI, N. PG, O. CE, P. COH, Q. DG. Color key: x-m-, brown column; x-m+, blue column; x+m-, red column; x+m+, green column. “*” = p < 0.05; “**” = p < 0.01 [file 40170_2022_283_MOESM1_ESM.tif]

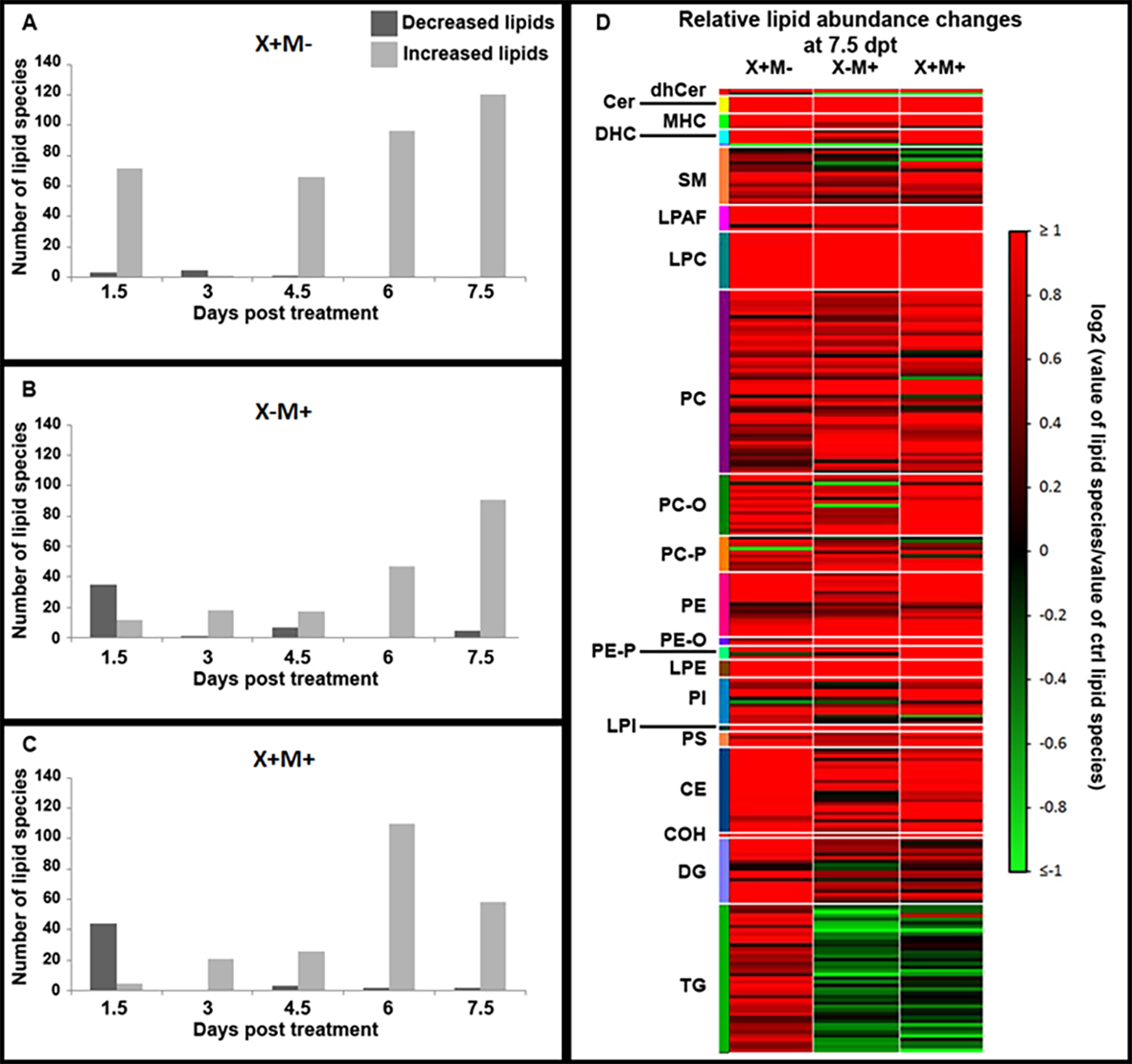

Supplement: Supplementary file 2 — Additional file 2: Supplementary Figure 2. Expression profiles for lipid species after transgene induction. A-C. Total number of lipid species showing a decrease (dark gray) or an increase (light gray) in all transgene types from 1.5 to 7.5 dpt. D. Heat map showing all lipid species detected at 7.5 dpt in all transgene types compared to DOX only (no transgene) control liver tissues. A red band shows an increase of a particular lipid species in cancer cells, a green band shows a decrease of a particular lipid species, and a dark band indicated no change. Data expressed as log2 transformation of the value of lipid species/value of control lipid species. [file 40170_2022_283_MOESM2_ESM.tif]

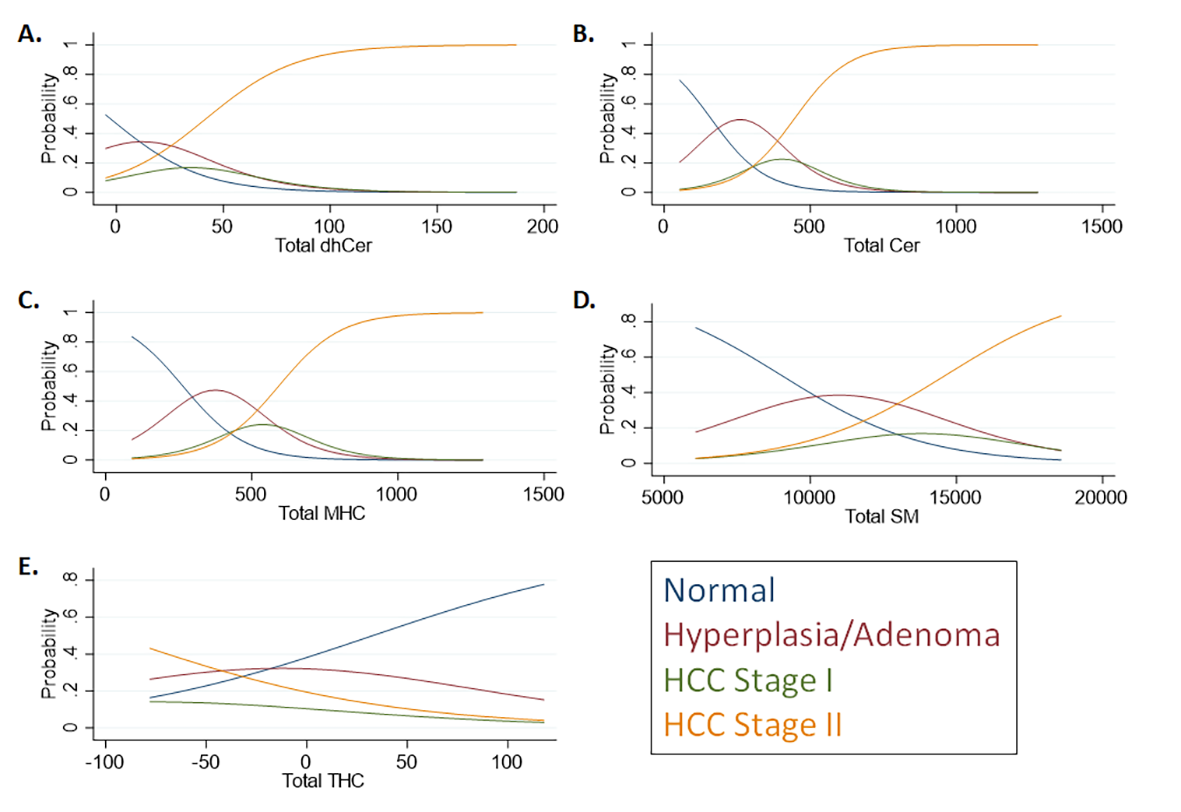

Supplement: Supplementary file 3 — Additional file 3: Supplementary Figure 3. Univariate modelling analysis for dhCer, Cer, MHC, THC and SM lipid classes. Several lipid classes analyzed with univariate modelling analysis were not associated with HCC disease progression by MFP analysis. A. dhCer, B. Cer, C. MHC, D. THC, E. SM. X-axis units: pmol/mg. Key: “OR” (odds ratio); dark blue lines (normal); dark red lines (hyperplasia/ adenoma); green lines (HCC stage I); orange lines (HCC stage II). [file 40170_2022_283_MOESM3_ESM.tif]

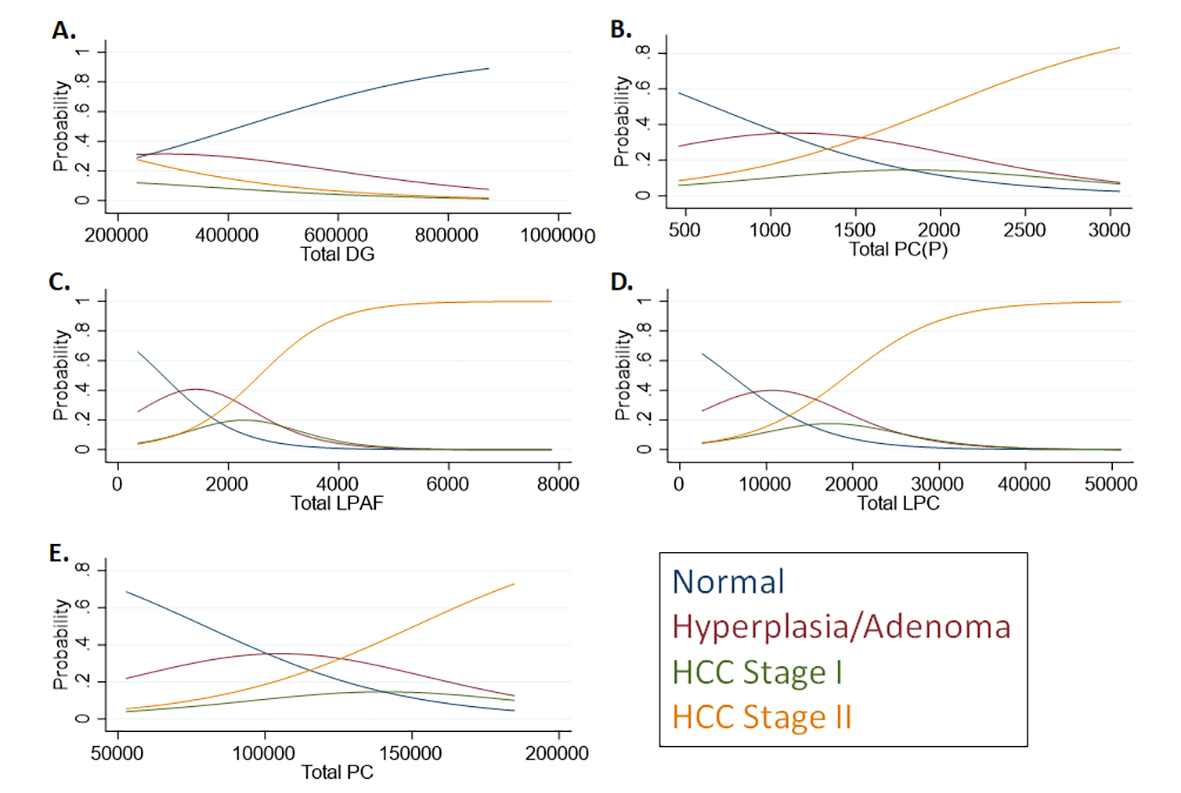

Supplement: Supplementary file 4 — Additional file 4: Supplementary Figure 4. Univariate modelling analysis for DG, PC(P), LPAF, LPC and PC lipid classes. Several lipid classes analyzed with univariate modelling analysis were not associated with HCC disease progression by MFP analysis. A. DG, B. PC(P), C. LPAF, D. LPC, E. PC. X-axis units: pmol/mg. Key: “OR” (odds ratio); dark blue lines (normal); dark red lines (hyperplasia/ adenoma); green lines (HCC stage I); orange lines (HCC stage II). [file 40170_2022_283_MOESM4_ESM.tif]

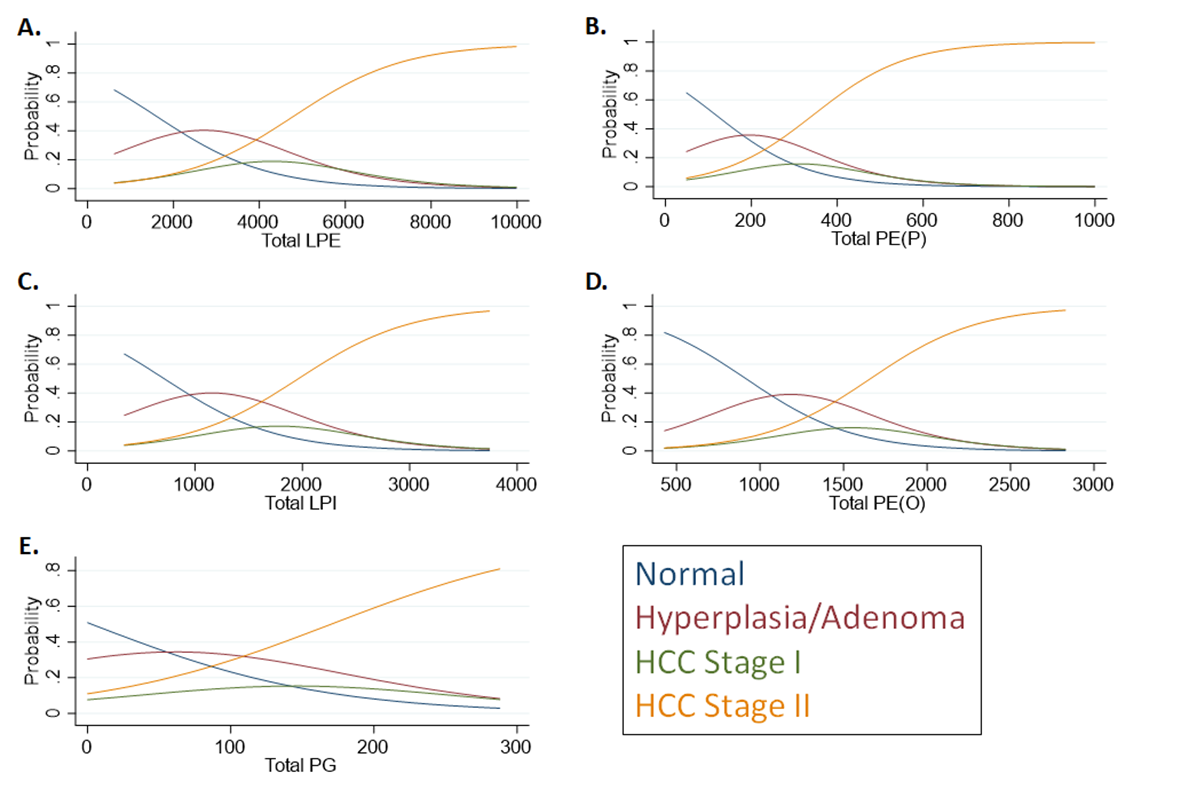

Supplement: Supplementary file 5 — Additional file 5: Supplementary Figure 5. Univariate modelling analysis for LPE, PE(P), LPI, PE(O) and PG lipid classes. Several lipid classes analyzed with univariate modelling analysis were not associated with HCC disease progression by MFP analysis. A. LPE, B. PE(P), C. LPI, D. PE(O), E. PG. X-axis units: pmol/mg. Key: “OR” (odds ratio); dark blue lines (normal); dark red lines (hyperplasia/ adenoma); green lines (HCC stage I); orange lines (HCC stage II). [file 40170_2022_283_MOESM5_ESM.tif]

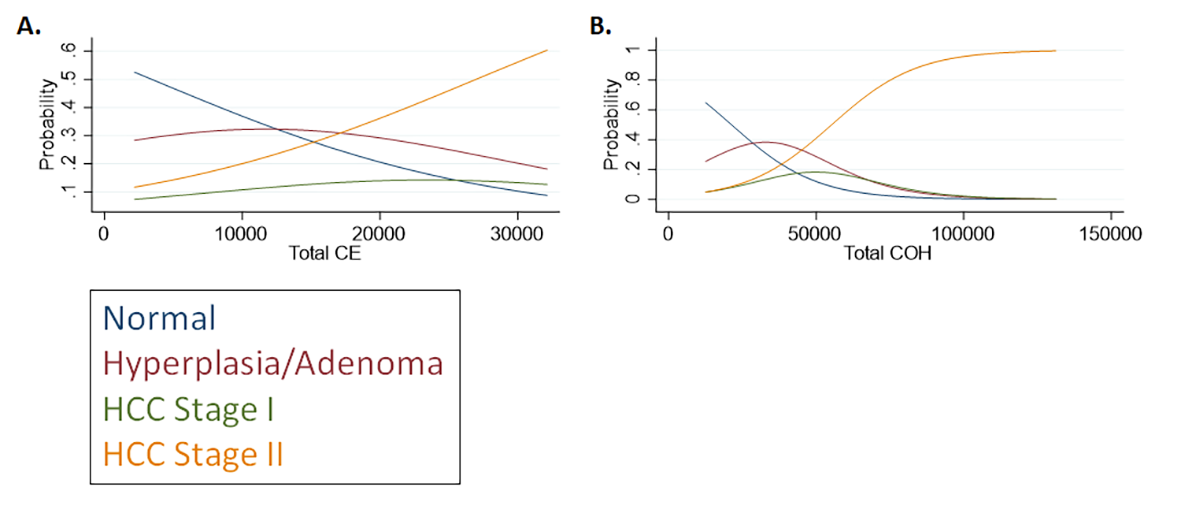

Supplement: Supplementary file 6 — Additional file 6: Supplementary Figure 6. Univariate modelling analysis for CE and COH lipid classes. Some lipid classes analyzed with univariate modelling analysis were not associated with HCC disease progression by MFP analysis. A. CE, B. COH. X-axis units: pmol/mg. Key: “OR” (odds ratio); dark blue lines (normal); dark red lines (hyperplasia/ adenoma); green lines (HCC stage I); orange lines (HCC stage II). [file 40170_2022_283_MOESM6_ESM.tif]
