# Supplementary material for: Identification of novel lipid biomarkers in xmrk- and Myc-induced models of hepatocellular carcinoma in zebrafish
Source: Cancer Metab. 2022 Apr 4;10:7. doi: 10.1186/s40170-022-00283-y (PMC8981695; doi:10.1186/s40170-022-00283-y)
Supplement: Supplementary file 7 — Additional file 7: Supplementary Table 1. List of qPCR primers. [file 40170_2022_283_MOESM7_ESM.docx]

| **Gene** | **Gene Name** | **Forward Primer** | **Reverse Primer** |
| --- | --- | --- | --- |
| *srebf1* | Sterol regulator element binding transcription factor 1 | CATCCACATGGCTCTGAGTG | CTCATCCACAAAGAAGCGGT |
| *chrebp* | Carbohydrate response element-binding protein | GGAGATGGACTCGCTCTTTG | GCAGAGGCTCAAAAGTGTCC |
| *cebpa* | CCAAT/enhancer-binding protein a | AACGGAGCGAGCTTGACTT | AAATCATGCCCATTAGCTGC |
| *fasn* | Fatty acid synthase | ATCTGTTCCTGTTCGATGGC | AGCATATCTCGGCTGACGTT |
| *agpat4* | 1-acyglycerol-3-phosphate O-acyltransferase 4 | TTGGCGAAAAAGGAACTGTC | GGTGGTACTTGAGTTTGGGG |
| *ppap2a* | Phosphatidic acide phosphatise type 2A | CAGTTCTTCCTGATTGCTGC | TCCTCAAAGCTTAGTTCGGG |
| *dgat2* | Diacylglycerol O-acyltransferase 2 | TGGGGCTTTTTGTAACTTCG | TCTTCCTGGTGCACAGTCC |
| *adipoqa* | adiponectin, C1Q and collagen domain containing, a | AGGATTTCCAGGCAAAAGAG | GCCATTGATGGTGAGGTGAT |
| *pparab* | peroxisome proliferator-activated receptor alpha b | CGTCGTCAGGTGTTTACGGT | AGGCACTTCTGGAATCGACA |
| *cpt1* | carnitine palmitoyltransferase 1A (liver) | ACTCTCGATGGACCCTGTGA | CTGGATGAAGGCATCTGGAC |
| *I-pbe* | enoyl-CoA, hydratase/3-hydroxyacyl CoA dehydrogenase | GGAAGGTGAGTGTGGCAGTT | GGAGGATCTTTGGGGTCAAC |
| *cyp4a10* | cytochrome P450, family 4, subfamily T, polypeptide 8 | CATTCTCACAACCACAGAAC | GAACTTCCCATTTATCAAGC |
| *acox3* | acyl-CoA oxidase 3, pristanoyl | AAGGACATCGAGCGAATGAT | CTATGAAAGAGTGGAGGCCG |
| *β-actin* | beta-actin |  |  |
